# High temporal frequency light response in mouse retina is mediated by ON and OFF bipolar cells and requires FAT3 signaling

**DOI:** 10.1101/2023.11.02.565326

**Published:** 2023-11-04

**Authors:** Evelyn C. Avilés, Sean K. Wang, Sarina Patel, Shuxiang Shi, Lucas Lin, Vladimir J. Kefalov, Lisa V. Goodrich, Constance L. Cepko, Yunlu Xue

**Affiliations:** 1. Department of Neurobiology, Blavatnik Institute, Harvard Medical School, Boston, MA 02115; 2. Present address: Facultad de Ciencias Biologicas, Pontificia Universidad Catolica deChile, Santiago, Chile; 3. Departments of Genetics and Ophthalmology, Harvard Medical School, Boston, MA 02115; 4. Howard Hughes Medical Institute, Boston, MA 02115; 5. Lingang Laboratory, Shanghai, China, 200031; 6. School of Life Science and Technology, ShanghaiTech University, Shanghai, China, 201210.; 7. Gavin Herbert Eye Institute & Center for Translational Vision Research, University of California, Irvine, CA 92697; 8. Lead contact

**Keywords:** high frequency vision, retinal physiology, FAT cadherins, bipolar cells, GRIK1, GRM6

## Abstract

Vision is initiated by the reception of light by photoreceptors and subsequent processing via parallel retinal circuits. Proper circuit organization depends on the multi-functional tissue polarity protein FAT3, which is required for amacrine cell connectivity and retinal lamination. Here we investigated the retinal function of *Fat3* mutant mice and found decreases in physiological and perceptual responses to high frequency flashes. These defects did not correlate with abnormal amacrine cell wiring, pointing instead to a role in bipolar cell subtypes that also express FAT3. Indeed, similar deficits were observed in mice lacking the bipolar cell glutamate receptors GRIK1 (OFF-bipolar cells) and GRM6 (ON-bipolar cells). Mechanistically, FAT3 binds to the synaptic protein PTPσ and is required to localize GRIK1 to OFF-cone bipolar cell synapses with cone photoreceptors. How FAT3 impacts ON-cone bipolar cell function at high temporal frequency remains to be uncovered. These findings expand the repertoire of FAT3’s functions and reveal the importance of both ON- and OFF-bipolar cells for high frequency light response.

## Introduction

**Figure 1:**
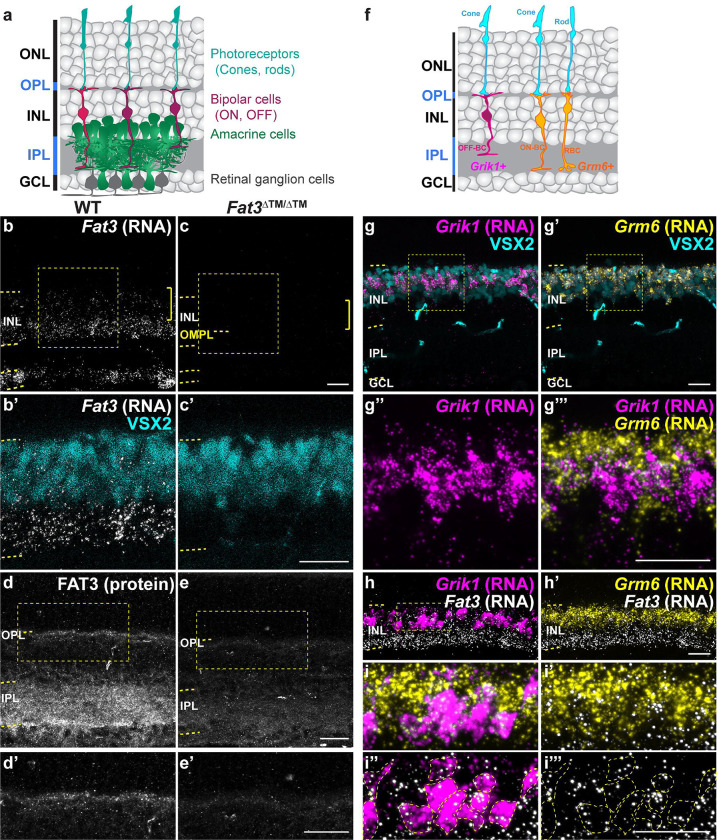
*Fat3* RNA is enriched in OFF-cone bipolar cells. (a) Schematic representation of retinal layers and their neurons. (b) *in situ* hybridization for *Fat3* RNA in WT P22 retinas. (c) *in situ* hybridization for *Fat3* RNA in *Fat3*^ΔTM/ΔTM^ P22 retinal tissue. In b and c the RNA puncta are shown in white and the yellow brackets indicate the area of VSX2+ cell bodies. Yellow dashed lines demark the inner nuclear layer (INL) and the outer misplaced plexiform layer (OMPL) in *Fat3*^ΔTM/ΔTM^ tissue. The squares demark the insets seen in b’ and c’ at higher magnification. VSX2 protein is seen in cyan. (d) Hybridization Chain Reaction-Immunohistochemistry (HCR-IHC) of FAT3 in wild type retinas. Inset demarked in a yellow box in d is shown at higher magnification in d’. (e) HCR-IHC of FAT3 in *Fat3*^ΔTM/ΔTM^ mutant retinas. Inset demarked in a yellow box in e is shown at higher magnification in e’. (f) Schematic representation of *Grik1* and *Grm6* RNA enrichment in bipolar cells, according to data in Figure S2. (g) *in situ* hybridization of *Grik1* RNA (magenta) and Grm6 RNA (yellow, g’) with immunostaining for VSX2 (cyan). The insets in g and g’ are shown at a higher magnification in g” and g”’. (h) Triple *in situ* hybridization to *Fat3*, *Grik1* and *Grm6* RNA. Inset in h is seen at higher magnification in i-i”’. (i) Higher magnification of inset shown in h. Yellow dashed lines in i” and i”’ demark *Grik1* RNA+ cell bodies. *Fat3* RNA (white) is shown together with *Grm6* RNA in i’, with *Grik1* RNA in i” and alone in i”’. Scale bars: 20μm.

The remarkable ability to detect light over a wide range of intensities and frequencies is accomplished by highly specialized cell types and circuits within the retina. These circuits transform signals originating from the photoreceptors following their initial synapse with bipolar cells (BCs), which are classified as ON or OFF depending upon their response to photoreceptor signals, setting the stage for downstream processing events. Critical to these transformations are the >80 types of retinal interneurons^1–4^, which are organized into laminae with the cell bodies of the horizontal, BCs, and amacrine cells (ACs) located in the inner nuclear layer (INL), while the photoreceptor cell bodies reside in the outer nuclear layer (ONL). Retinal interneurons form synaptic connections in two layers: in the outer plexiform layer (OPL) among BCs, horizontal cells and photoreceptors, and in the inner plexiform layer (IPL) among BCs, ACs, and the output neurons of the retina, the retinal ganglion cells (RGCs) (Figure 1a). Despite the stereotyped and conserved organization of retinal neurons and their connections, it is unclear how important the lamination is for the sense of vision. Also unknown is how the organization of most retinal circuits mediate specific visual responses, e.g. which cell types and connections are needed to process and transmit high frequency signals^5^.

Several features of retinal circuit assembly depend upon FAT3, a tissue polarity protein^6–8^. FAT cadherins are transmembrane receptors that can sense cell position in the environment *via* their huge extracellular domains. They induce appropriate changes in cell morphology *via* their intracellular domains, thereby creating cellular asymmetries that are aligned across the tissue. In *Fat3* mutant retinas, many ACs migrate to ectopic locations in the IPL and the ganglion cell layer (GCL). They also fail to retract their trailing neurites, which go on to form ectopic synapses in two misplaced plexiform layers, one in the INL (the outer misplaced plexiform layer, OMPL) and one below the GCL (the inner misplaced plexiform layer, IMPL)^6–8^. These ectopic layers contain synapses between ACs and between ACs and rod BCs, which do not express FAT3^2^, but seem to “follow” their AC partners to abnormal locations^7^. FAT3, which is localized to AC dendrites in the IPL, mediates these effects by localizing cytoskeletal effectors needed for migration and retraction^6^. The FAT3 intracellular domain also binds a variety of synaptic proteins^6^ and is therefore poised to control synapse localization or function independent of its effects on cell morphology. A direct functional role at the synapse has not been described, and it was not clear how the loss of FAT3 impacts vision.

Here, we used retinal physiology and behavioral analyses to investigate the effects of FAT3 disruptions on retinal function. We demonstrate that FAT3 ensures formation of synapses between cones and BC dendrites that are required for proper transmission of high temporal frequency signals. Basic light responses of retinal neurons were found to be preserved in *Fat3* mutant mice despite the highly abnormal ectopic plexiform layers. However, the overall retinal response to 30 Hz flashes were severely reduced in amplitude and *Fat3* mutants behaved as if they see constant illumination. Analysis of a variety of conditional *Fat3* knock-out mice revealed that this phenotype is not due to altered AC lamination. Instead, we found that the OFF-cone pathway is abnormal, as revealed by an *in vivo* electroretinogram (ERG) protocol that we developed. Loss of *Grik1*, which encodes an ionotropic glutamate receptor specifically localized to OFF-Cone BC (CBC) dendrites^9,10^, showed a reduction in the d-wave, but had little effect on the amplitude of the 30 Hz ERG. Indeed, only the loss of *Grik1* along with *Grm6,* which encodes a metabotropic glutamate receptor in ON-CBCs, resulted in reductions in the amplitude of the ERG response to high frequency flickering lights, indicating that both CBC pathways are required for high temporal frequency light responses. Further, we show that the FAT3 intracellular domain binds to the synaptic protein PTPσ, and that in *Fat3* mutants, both PTPσ and GRIK1 are present at reduced levels in the ribbon synapses that link cones to OFF-CBCs. Thus, FAT3 is required to set up and/or maintain a properly organized and functional synapse between BCs and photoreceptors and the transmission of high frequency signals, highlighting its multiple and versatile roles in the development and function of retinal circuitry.

## Results

### Global loss of *Fat3* affects the response of the cone pathway to high temporal frequency light

In *Fat3*^ΔTM/ΔTM^ mutant mice, which lack a membrane localized form of FAT3, retinal lamination is strongly disrupted, with changes in the position of ACs and their synapses with other ACs, as well as with other retinal cell types^7^. The abnormal lamination can also be seen by optical coherence tomography (OCT) imaging in animals *in vivo* (Figure S1a). To assess the functional consequences of this change in circuit organization, we used ERG, a common way to measure electrical changes in the retina in response to light. By altering the stimulus, it is possible to reveal the contributions of specific cell types. For instance, signaling through the rod pathway is assayed by performing the ERG after dark-adaptation and under scotopic conditions, using dim flashes that elicit little response from the cone pathway. Conversely, photopic ERG tests isolate the cone pathway by light-adapting the eyes and using a background light that saturates rod phototransduction. In an ERG waveform, the a-wave originates from photoreceptors and is followed by the b-wave, which reflects the activity of rod bipolar cells (RBC) and/or ON-types of CBCs. By flashing the stimulus on and off with increasing frequency, it is possible to determine how well photoreceptors and BCs are able to resolve temporal differences in the visual stimulus. As the frequency of the flash increases, OFF-CBCs are hypothesized to dominate the response based on evidence from a few genetically modified strains^11^. Additionally, the ERG d-wave, which emerges when turning off a light step that lasts for a few seconds^12^, is thought to represent mainly OFF-CBC activity^13^. However, definitive *in vivo* evidence is lacking, and d-wave measurements are not usually collected in the lab or the clinic.

Conventional ERG assays under scotopic and photopic conditions revealed no obvious difference in *Fat3*^ΔTM/ΔTM^ vs. *Fat3*^ΔTM/+^ littermates, indicating that overall signaling through rod and cone pathways was preserved (Figure S1b–e). Likewise, the oscillatory potentials between the a- and b-waves, which are thought to reflect the activity of ACs and/or RGCs^14^, showed no significant change in the number, amplitude or timing of peaks (Figure S1f). Thus, *Fat3*^ΔTM/ΔTM^ mutant mice can detect standard light stimuli despite changes in retinal lamination.

Although previous work showed its role in ACs^6,7^, *Fat3* is also expressed in RGCs and some subtypes of BCs, especially OFF-CBCs^2^ (Figure 1b,c, Figure S2). Consistent with this expression pattern, FAT3 protein localizes not only to the IPL but also to the OPL, where BC dendrites form synapses with photoreceptors (Figure 1d), raising the possibility of additional effects on retinal function. Indeed, *in situ* hybridization confirmed co-expression of *Fat3* with the OFF-CBC marker *Grik1,* which encodes an ionotropic glutamate receptor^2,15^(Figure 1f–i. *Fat3* is also expressed in some *Grik1*-negative CBCs, which are positive for the ON-CBC marker *Grm6*, though to a less degree (Figure 1i, S2). This suggested that FAT3 might be required for retinal functions that would not be detected using standard ERG, as conventional photopic and scotopic ERG measures overall activities from populations of photoreceptors and the ON-pathway, but not the OFF-pathway or an individual ON-CBC subtype.

**Figure 2:**
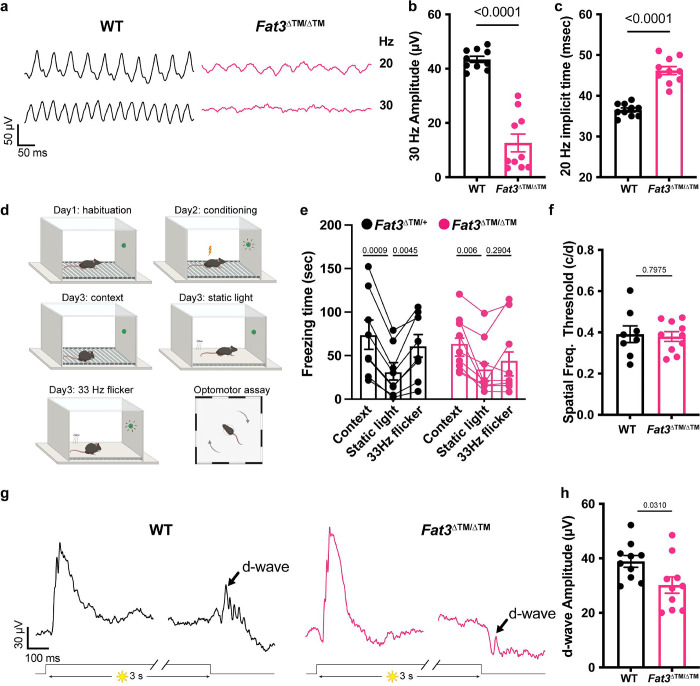
Flicker ERG and vision at high frequency and step ERG of *Fat3*-deficient mice. (a) Representative flicker ERG raw traces of WT control and *Fat3*^ΔTM/ΔTM^ eyes elicited by 3.162 cd s/m^2^ flashes at 20 and 30 Hz frequencies. (b) Flicker ERG amplitude at 30 Hz for WT control (n=10) and *Fat3*^ΔTM/ΔTM^ (n=10) eyes. Unpaired two-tailed Student’s t test. (c) Flicker ERG implicit time (1^st^ peak) at 20 Hz for WT control (n=10) and *Fat3*^ΔTM/ΔTM^ (n=10) eyes. Unpaired two-tailed Student’s t test. (d) Schematics of fear conditioning and optomotor behavioral experiment. On Day 1, a mouse is brought to the electric-shock cage with a floor of metal bars for habituation of the environment. On Day 2, the mouse is conditioned by electrical shock paired with 33 Hz flashing light. On Day 3 (see Supplementary movies for representative recordings from Fat3 mutant mice), the mouse is first subjected to a contextual check, in which the “Context” measures the freezing time of the mouse after it is brought back to the electric shock cage, which presents a fear-associated context environment, without the shock. “Static” measures the freezing time of the mouse with a static light, after the covering the metal bars and an odor change. Following this measurement, a 33 Hz flickering light is turned on, and the freezing time of the mouse is measured, as the “flicker” time. (e) Fear conditioning responses as freezing time (sec) from *Fat3*^ΔTM/+^ (n=8) and *Fat3*^ΔTM/ΔTM^ (n=9) mice. One-way ANOVA with Dunnett multiple comparison test. (f) The visual threshold of spatial frequency of WT (n=8) and *Fat3*^ΔTM/ΔTM^ (n=10) mice measured with the optomotor behavioral assay shown in the bottom cartoon in panel d. Unpaired two-tailed Student’s t test. (g) Representative step ERG raw traces of WT (n=10) control and *Fat3*^ΔTM/ΔTM^ (n=10) eyes elicited by a 3-second step light at 1000 cd/m^2^ intensity. (h) Statistics of step ERG amplitudes (b-wave, d-wave and b : d ratio) of WT (n=10) control and *Fat3*^ΔTM/ΔTM^ (n=10) eyes elicited by a 3-second step of light at 1000 cd/m^2^ intensity. Unpaired two-tailed Student’s t test.

To determine whether *Fat3* is required for OFF-CBC function *in vivo*, we recorded ERGs in response to lights that flicker in the high frequency range (i.e. over 15 Hz, as defined by Seeliger and colleagues^11^). Flicker ERG responses in this range are hypothesized to be dominated by the OFF-pathway but direct evidence has been lacking^11^. We observed a significant decrease in the amplitude of responses to lights flickering at 30 Hz in *Fat3*^ΔTM/ΔTM^ compared to their *Fat3*^ΔTM/+^ littermates (Figure 2a,b). Additionally, the timing of the first peak in response to 20 Hz stimulation (“the implicit time”) was delayed (Figure 2c). 30 Hz implicit time was not measured, as many *Fat3* mutant mice presented no response at this frequency. These results suggested that the transmission of high temporal frequency light responses was impaired, and, more specifically, in BCs, where the signal to the flicker ERG orignates^11^. To determine if this physiological deficit in the retina had perceptual consequences, behavioral assays were conducted, using contextual and vision-cued fear conditioning tests^16,17^. Normally, fear conditioned animals increase the time of “freezing” if they have been conditioned to associate an unpleasant stimulus with an environmental cue, in this case, 33 Hz light (Figure 2d, **Supplementary movies**). In contrast to *Fat3*^ΔTM/+^ mice (n=8 animals), some *Fat3*^ΔTM/ΔTM^ mice (5 out of 9 animals) failed to show a freezing response when switching from a static light to a 33 Hz flicker (Figure 2e). The lack of response was not due to an inability to form fear memories, as all *Fat3*^ΔTM/ΔTM^ and *Fat3*^ΔTM/+^ animals froze less when switching from an unpleasant olfactory and tactile context (group “context”) to a novel and safe context within the group with static light (group “static light”) (Figure 2e). Mutant mice also performed like the WT controls in an optomotor behavioral assay, which measures spatial discrimination (Figure 2f).

Responses to different frequencies of light are used to study the temporal properties of vision at photoreceptor, brain or psychophysical levels^18^. The flicker ERG at high temporal frequency was proposed, but had not been established, as a test for OFF-CBC function. Usually, OFF-CBC function is probed using patch-clamping of single cells, or assessed at the population level by examination of the d-wave of the ERG. To assay overall function of the OFF-CBC population, we designed an *in vivo* ERG protocol for mice based on a study of the *ex vivo* ERG response of amphibian retinas. This assay measures the retinal voltage change, the d-wave, after turning off a long step of light^12^, which is called a “step ERG”. Using this newly developed *in vivo* protocol on mice, we found that WT mice had a strong d-wave with robust oscillatory potentials at the end of a three-second exposure to a 1,000 cd/m^2^ step of light (Figure 2g). By contrast, in *Fat3*^ΔTM/ΔTM^ mice, the d-wave amplitude was decreased, and the d-wave related oscillatory potentials were absent (Figure 2g,h). Thus, responses to both high frequency flickering light and the light being turned off showed that *Fat3*^ΔTM/ΔTM^ mice have deficits in processing specific types of visual stimuli.

### Retinal lamination defects do not disrupt responses to flickering stimuli

**Figure 3:**
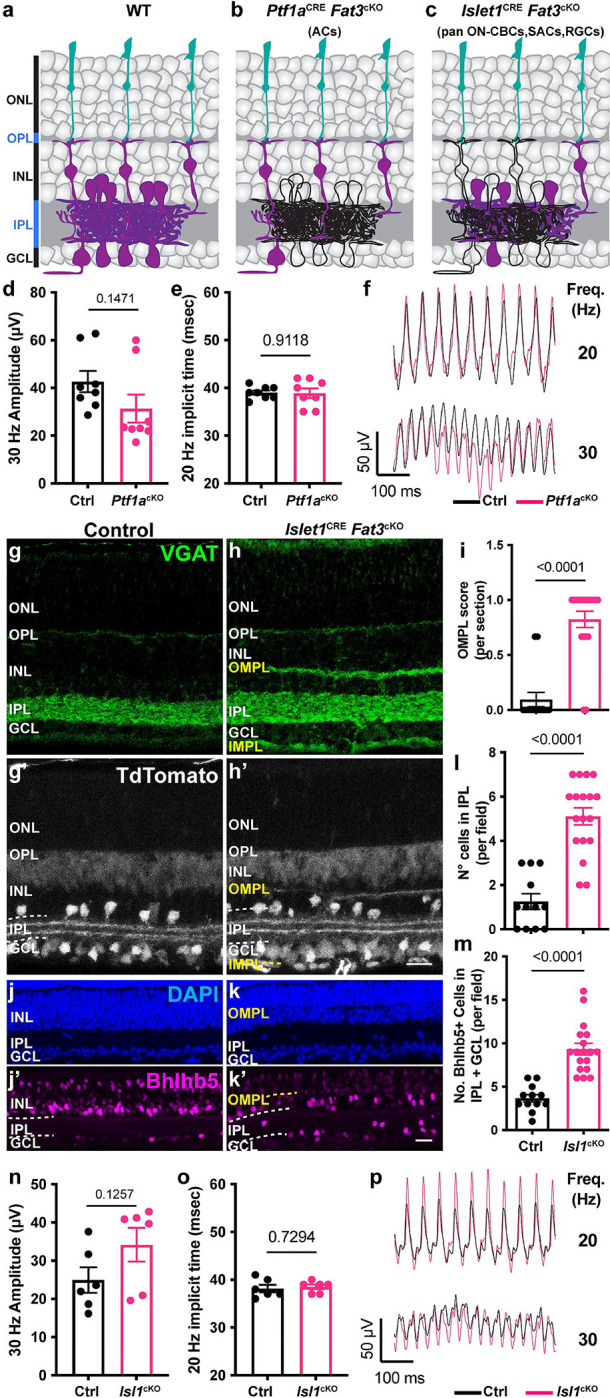
Flicker ERG at high frequency of *Ptf1a*^CRE^ and *Isl1*^CRE^ conditional *Fat3* mice. (a) Schematic representation of cell classes that express FAT3 in wild type tissue. Cells that express FAT3 are represented in magenta. (b) Schematic representation of cell classes, i.e. ACs, that lose *Fat3* expression in a *Ptf1a*^CRE^ cKO, shown in black outlines. (c) Schematic representation of cell classes, i.e. starburst ACs, RGCs and ON-CBCs, that lose *Fat3* expression in an *Isl1*^CRE^ cKO, shown in black outlines. (d) Flicker ERG amplitude at 30 Hz for the *Ptf1a*^CRE^ *Fat3*cKO condition. Control genotypes are *Ptf1a*^CRE^;*Fat3*^fl/+^ (n=8 eyes) and *Ptf1a*^cKO^ genotypes are *Ptf1a*^CRE^;*Fat3*^fl/ΔTM^ (n=8 eyes). Unpaired two-tailed Student’s t test. (e) Flicker ERG implicit time at 20 Hz for *Ptf1a*^CRE^;*Fat3*^fl/+^ (control, n=8 eyes) and *Ptf1a*^CRE^;*Fat3*^fl/ΔTM^ (*Ptf1a*^cKO^, n=8 eyes). Unpaired two-tailed Student’s t test. (f) Representative flicker ERG raw traces for *Ptf1a*^CRE^;*Fat3*^fl/+^ (control, n=8 eyes) and *Ptf1a*^CRE^;*Fat3*^fl/ΔTM^ (*Ptf1a*^cKO^, n=8 eyes). (g) VGAT immunostaining for control *Isl1*^CRE/+^;*Fat3*^fl/+^ mice. (h) VGAT immunostaining for *Isl1*^CRE/+^;*Fat3*^fl/ΔTM^ *Isl1*^cKO^. TdTomato reporter of Cre expression is seen in g’-h’. (i) Quantification of the OMPL score for *Isl1*^CRE^ *Fat3*^cKO^. Controls (*Isl1*^CRE/+^;*Fat3*^fl/+^): 0.095 ± 0.065 (n=14 sections, N=3 animals); *Isl1*^cKO^ (*Isl1*^CRE/+^;*Fat3*^fl/ΔTM^): 0.825 ± 0.074 (n=19 sections, N=3 animals), Mann-Whitney test. (j) DAPI and Bhlhb5 immunostaining of control retinas (*Isl1*^CRE/+^;*Fat3*^fl/+^). (k) DAPI and Bhlhb5 immunostaining of *Isl1*^CRE^ *Fat3*^cKO^ (*Isl1*^CRE/+^;*Fat3*^fl/ΔTM^) retinas. (l) Quantification of the number of nuclei per field in the IPL. Controls (*Isl1*^CRE/+^;*Fat3*^fl/+^): 1.25 ± 0.35 (n=12 sections, N=3 animals); *Isl1*^cKO^ (*Isl1*^CRE/+^;*Fat3*^fl/ΔTM^): 5.11 ± 0.39 (n=18 sections, N=3 animals). *t* test. (m) Quantification of the number of Bhlhb5+ nuclei per field in the IPL and GCL. Controls (*Isl1*^CRE/+^;*Fat3*^fl/+^): 3.67 ± 0.43 (n=12 sections, N=3 animals); *Isl1*^cKO^ (*Isl1*^CRE/+^;*Fat3*^fl/ΔTM^): 9.33 ± 0.67 (n=18 sections, N=3 animals). *t* test. (n) Flicker ERG amplitude at 30 Hz for control (*Isl1*^CRE/+^;*Fat3*^fl/+^, n=6 eyes) and *Isl1*^cKO^ (*Isl1*^CRE/+^;*Fat3*^fl/ΔTM^, n=6 eyes). Unpaired two-tailed Student’s t test. (o) Flicker ERG implicit time at 20 Hz for control (*Isl1*^CRE/+^;*Fat3*^fl/+^, n=6 eyes) and *Isl1*^cKO^ (*Isl1*^CRE/+^;*Fat3*^fl/ΔTM^, n=6 eyes). Unpaired two-tailed Student’s t test. (p) Representative flicker ERG raw traces for control and *Isl1*^CRE^ *Fat3*cKO. Scale bars: 20μm.

We next asked which changes to retinal organization and function are responsible for the impaired high frequency light response in *Fat3*^ΔTM/ΔTM^ mutant mice. To test the impact of an ectopic plexiform layer, *Fat3* was deleted specifically from ACs using *Ptf1a*^CRE 7,19^ and a *Fat3* floxed allele (Figure 3a,b). In these conditional knock-out (cKO) mutants, ACs migrate normally, but do not retract their trailing processes, leading to formation of an OMPL^7^. We found that the 30 Hz flicker ERG amplitude and 20 Hz implicit time were normal in *Ptf1a*^CRE^ *Fat3*cKO mice compared to littermate *Ptf1a*^CRE^ control mice (Figure 3d–f), indicating that the high frequency light response defects were not secondary either to the presence of *Fat3* mutant ACs or the lamination defects they caused. To survey other FAT3+ cells for possible effects on high frequency light response, we used the *Isl1*^CRE^ mouse line to drive recombination in all ON-CBCs, starburst ACs, and RGCs, but not in OFF-CBCs^20^ (Figure 3c,g,h). Although *Isl1*^CRE^ *Fat3*cKO mice exhibited all of the cellular phenotypes previously described in the retina of *Fat3*^ΔTM/ΔTM^ mice (Figure 3g–m), the 30 Hz flicker ERG amplitude and 20 Hz implicit time were normal compared to littermate *Isl1*^CRE^ control mice (Figure 3n–p). Thus, the high frequency light response defects are not due to the gross disorganization of retinal lamination and do not reflect a role for FAT3 in RGCs or ON-CBCs alone. The 30 Hz amplitude and 20 Hz implicit timing were also unaffected by removal of *Fat3* from GABAergic ACs and the type 2 OFF-CBC subset using *Bhlhe22*^CRE^ (Figure S3a–k) or loss of non-type 2 OFF-CBCs, as occurs in *Fezf2*^−/−^ mutants^2,21^ (Figure S3l–q). We were not able to directly test whether high frequency light response requires FAT3 in OFF-CBCs, as there is no Cre line that is active in all OFF-CBCs. Nonetheless, these experiments showed that the high frequency light response defects are not caused by retinal lamination defects and most likely involve the loss of FAT3 from OFF-CBCs, potentially in combination with loss from other cell types.

### FAT3 intracellular signaling is critical for high frequency light response and the step ERG d-wave

**Figure 4:**
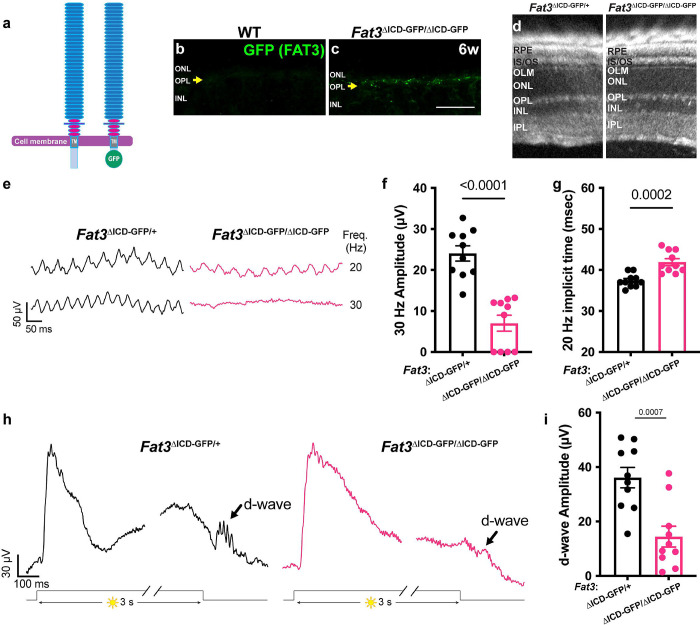
High frequency flicker ERG and step ERG of FAT3 intracellular domain (ICD) deficient mice. (a) Schematics of molecular structure of FAT3 wild type protein and FAT^ΔICD-GFP^. (b) Immunostaining for GFP in WT retinal sections. The arrow points the OPL. (c) Immunostaining for GFP in *Fat3*^ΔICD-GFP/ΔICD-GFP^ retinal sections. The arrow points the OPL. (d) Representative OCT images of *Fat3*^ΔICD-GFP/+^ control and *Fat3*^ΔICD-GFP/ΔICD-GFP^ eyes. (e) Representative flicker ERG raw traces of *Fat3*^ΔICD-GFP/+^ control and *Fat3*^ΔICD-GFP/ΔICD-GFP^ eyes elicited by 3.162 cd s/m^2^ flashes at 20 and 30 Hz frequencies. (f) Flicker ERG amplitude at 30 Hz for *Fat3*^ΔICD-GFP/+^ control (n=10 eyes) and *Fat3*^ΔICD-GFP/ΔICD-GFP^ (n=10) eyes. Unpaired two-tailed Student’s t test. (g) Flicker ERG implicit time at 20 Hz for *Fat3*^ΔICD-GFP/+^ control (n=10 eyes) and *Fat3*^ΔICD-GFP/ΔICD-GFP^ (n=10) eyes at 20 Hz. Unpaired two-tailed Student’s t test. (h) Representative step ERG raw traces of *Fat3*^ΔICD/+^ control and *Fat3*^ΔICD-GFP/ΔICD-GFP^ eyes elicited by a 3-second step light at 1000 cd/m^2^ intensity. (i) Statistics of step ERG d-wave amplitudes for *Fat3*^ΔICD-GFP/+^ control (n=10 eyes) and *Fat3*^ΔICD-GFP/ΔICD-GFP^ (n=10) eyes elicited by a 3-second step of light at 1000 cd/m^2^ intensity. Unpaired two-tailed Student’s t test.

FAT3 is a versatile protein that can mediate non-autonomous interactions via its extracellular domain and autonomous effects by recruiting different sets of cytoskeletal effectors to its ICD^6^. To better understand how FAT3 signaling supports high frequency visual signal transmission and contributes to the step ERG d-wave, we analyzed *Fat3*^ΔICD-GFP^ animals, in which most of the ICD is replaced with GFP while keeping the extracellular and transmembrane domains anchored to the cell membrane^6^ (Figure 4a). These animals showed expression of the FAT3-GFP fusion protein in the OPL (Figure 4b,c), consistent with FAT3 protein localization (Figure 1d). In *Fat3*^ΔICD-GFP/ΔICD-GFP^ mutant mice, ACs migrate to abnormal cell layers and fail to retract their neurites, but do not form ectopic plexiform layers, as shown previously^6^. Therefore, analyzing *Fat3*^ΔICD-GFP/ΔICD-GFP^ mutants can reveal whether high frequency light response depends on intracellular signaling and/or the ability to form synapses.

Consistent with previous histological analysis^6^, no ectopic plexiform layers were detected by OCT *in vivo* imaging of *Fat3*^ΔICD-GFP/ΔICD-GFP^ eyes (Figure 4d). Nonetheless, the amplitude of the 30 Hz flicker ERG response was decreased in *Fat3*^ΔICD-GFP/ΔICD-GFP^ mice, as in *Fat3*^ΔTM/ΔTM^ mice (Figure 4e–f). Likewise, the 20 Hz implicit time was delayed and the amplitude of the d-wave in step ERG responses was decreased (Figure 4g–i). The loss of high frequency flicker ERG responses even in the absence of ectopic plexiform layers further underscores that changes in AC wiring do not contribute to altered visual function in *Fat3* mutant mice. Rather, these results suggest a potential role for FAT3 signaling in OFF-CBCs, in keeping with the results of the genetic analyses described above.

### Disruption of glutamate receptor signaling in CBCs alters ERG responses

**Figure 5:**
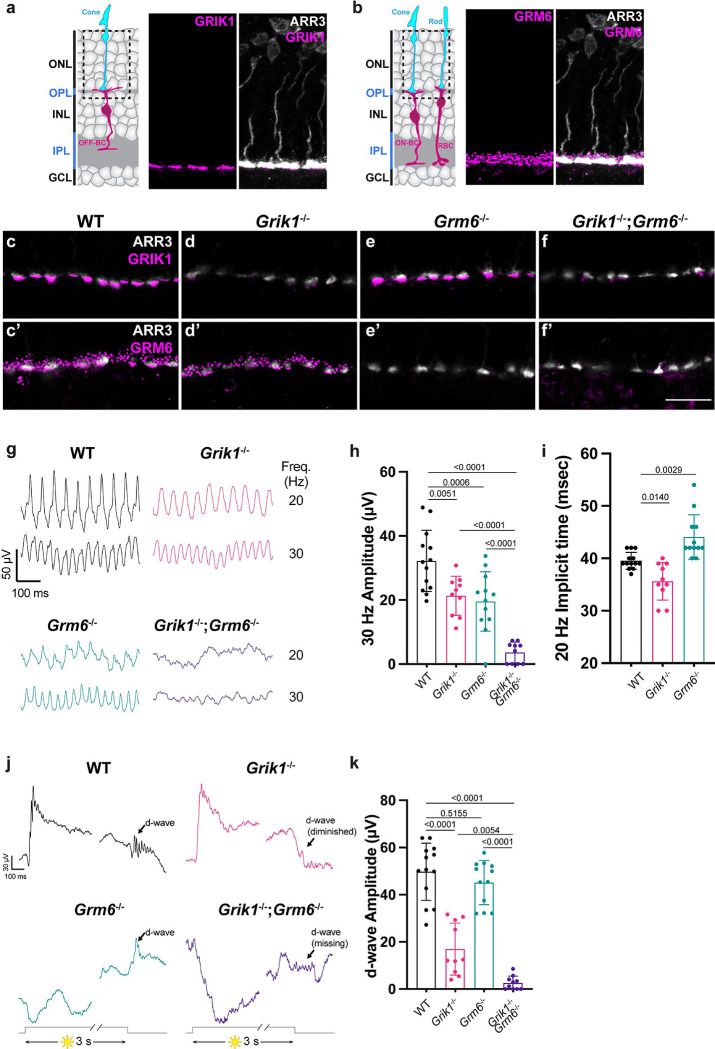
Immunostaining, high frequency flicker ERG and step ERG of mice lacking glutamate receptors, GRIK1 and GRM6. (a) Schematic representation of OFF-BCs and their synapses with cone photoreceptors. ARR3 (white) labels cones and GRIK1 (magenta) labels postsynaptic BC dendrites. (b) Schematic representation of ON-BCs and their synapses with cone photoreceptors. ARR3 (white) labels cones and GRM6 (magenta) labels postsynaptic cone and rod BC (RBC) dendrites. (c) GRIK1 and GRM6 immunostaining of adult WT retina. (d) GRIK1 and GRM6 immunostaining of *Grik1*^−/−^ retina. (e) GRIK1 and GRM6 immunostaining of *Grm6*^−/−^ retina. (f) GRIK1 and GRM6 immunostaining of *Grik1*^−/−^ *Grm6*^−/−^. White: ARR3, magenta: GRIK1 and GRM6. (g) Representative flicker ERG raw traces of WT, *Grik1*^−/−^, *Grm6*^−/−^ and *Grik1*^−/−^ *Grm6*^−/−^ eyes at 20 and 30 Hz frequencies. (h) Flicker ERG amplitude at 30 Hz for WT (n=13 eyes), *Grik1*^−/−^ (n=10 eyes), *Grm6*^−/−^ (n=12 eyes) and *Grik1*^−/−^;*Grm6*^−/−^ (n=10) eyes. One-way ANOVA with Dunnett multiple comparison test. (i) Flicker ERG implicit time at 20 Hz for WT (n=13 eyes), *Grik1*^−/−^ (n=10 eyes), *Grm6*^−/−^ (n=12 eyes) and *Grik1*^−/−^;*Grm6*^−/−^ (n=10) eyes. One-way ANOVA with Dunnett multiple comparison test. (j) Representative step ERG raw traces of WT, *Grik1*^−/−^, *Grm6*^−/−^ and *Grik1*^−/−^ *Grm6*^−/−^ eyes elicited by a 3-second step light at 1000 cd/m^2^ intensity. (k) Ensemble-averaged flicker d-wave of step ERG amplitudes of WT (n=13 eyes), *Grik1*^−/−^ (n=10 eyes), *Grm6*^−/−^ (n=12 eyes) and *Grik1*^−/−^;*Grm6*^−/−^ (n=10) eyes elicited by a 3-second step of light-OFF at 1000 cd/m^2^ intensity. One-way ANOVA with Dunnett multiple comparison test.

The above results suggest that the OFF-CBC pathway is likely to be essential for retinal responses to high frequency stimuli. To test this idea independently, we asked whether ERG responses also are altered upon loss of *Grik1*, which is the only subunit of ionotropic glutamate receptors (iGluR) enriched specifically in OFF-CBCs ^2,15,22,23^ (Figure S2, Figure 5a,c,d). To probe OFF-CBC morphology, we created a novel AAV vector (AAV8-Grik1-GFP) using our previously described *Grik1* enhancer/promoter element^15^. Viral delivery of the Grik1-GFP reporter resulted in the expression of GFP in OFF-CBCs, consistent with the *Grik1* expression profile, and revealed normal cellular morphologies in *Grik1*^−/−^ compared to WT littermates (Figure S4a,b). The 0.5 Hz and 10 Hz flicker ERG responses of *Grik1*^−/−^ were similar to WT littermates (Figure S4e–h). The amplitude of the response to 30 Hz flickering stimuli was decreased in *Grik1*^−/−^ mice (21.34 ± 1.92 μV, n=10 eyes vs. 32.23 ± 2.65 μV in WT, n=13 eyes, Figure 5g–i, S4i–k), but more modestly than in *Fat3* mutants (12.65 ± 3.29 μV vs. 43.43 ± 1.16 μV in WT; n=10 eyes each, Figure 2b). Additionally, the 20 Hz implicit time was earlier in *Grik1* mutants (35.60 ± 1.13 msec, n=10 eyes vs. 39.54 ± 0.45 msec in WT, n=13 eyes, Figure 5g,i, S4i,k), whereas this response was delayed in *Fat3* mutants (46.20 ± 1.00 msec, n=10 eyes vs. 36.50 ± 0.50 msec in WT, n=10 eyes; Figure 2c). Like *Fat3* mutants, *Grik1*^−/−^ mice showed decreased d-wave amplitudes and no d-wave oscillatory potentials in response to step stimuli when compared to WT littermates (Figure 5j,k,S4c,d).

As GRIK1 is the only iGluR subunit that is specifically expressed in OFF-CBCs and *Grik1*^−/−^ eyes did not completely lose the flicker ERG response or the d-wave, we wondered if glutamate receptors specific to ON-CBCs also contribute to the high frequency ERG response. Since the mGluR subunit, GRM6, is predominantly expressed by ON-CBCs and the loss of GRM6 is sufficient to remove all the transient ON-BC responses such as the ERG b-wave^2,24^ (Figure S2, Figure 5b), we generated *Grik1*^−/−^; *Grm6*^−/−^ double homozygous knockout mice and confirmed the deletion of both proteins with antibody staining (Figure 5c–f)^25,26^. The effect on the ERG response to 30 Hz flickering stimuli was greater in *Grm6*^−/−^;*Grik1*^−/−^ than *Grik1*^−/−^ mice, with strongly reduced amplitudes (3.63 ± 1.03 μV, n=10 eyes vs. 21.34 ± 1.92 μV in *Grik1*^−/−^, n=10 eyes, Figure 5h), similar to *Fat3* mutants (12.65 ± 3.29 μV in *Fat3*^ΔTM/ΔTM^, n=10 eyes, Figure 2b). Additionally, the 20 Hz implicit time was significantly delayed in *Grm6*^−/−^ mice (44.08 ± 1.22 msec, n=12 eyes vs. 39.54 ± 0.45 msec in WT, n=13 eyes, Figure 5i), similar to *Fat3* mutants (46.20 ± 1.00 msec, in *Fat3*^ΔTM/ΔTM^, n=10 eyes, Figure 2c). Thus, both GRIK1+ OFF-CBCs and GRM6+ ON-BCs contribute to retinal responses to high frequency light, despite the observation that *Fat3* mRNA was expressed with *Grm6* in only a few ON-CBC subtypes^2^ (Figure 1h’,i’, S2). Contrary to the assumption that the end of the step ERG is mediated entirely by OFF-CBCs, ERG recordings revealed a complete loss of the d-wave in *Grm6*^−/−^;*Grik1*^−/−^ mice, suggesting that both GRM6 and GRIK1 mediate this response *in vivo* (Figure 5j,k, S4c,d).

### GRIK1 localization to the ribbon synapse is reduced in *Fat3* mutants

To investigate possible cellular origins of the visual deficits, we analyzed the retina for possible changes in cones and BCs. We found no change in the number or distribution of cones (27.77 ± 0.91 cones, n=13 sections from 4 eyes vs. 29.00 ± 0.59 cones, n=15 sections from 4 WT eyes) or BCs (detected by staining for VSX2), which occupied a similar area in WT compared to *Fat3*^ΔTM/ΔTM^ mutant retinas (21.09 ± 1.04 arbitrary units, n=15 sections from 4 eyes vs. 21.59 ± 0.97 arbitrary units, n=15 sections from 4 WT eyes (Figure S5a–f). To visualize OFF-CBC morphology, WT and mutant P2/P3 retinas were injected with AAV8-Grik1-GFP and evaluated at P22. Whereas 100% (n=12 cells from 5 animals) of OFF-CBC axons terminated and stratified in the OFF sublaminas of the IPL in WT animals, only 49 ± 17 % of the mutant OFF-CBC axons (n=7 animals, 22 cells) terminated properly with 56 ± 19 % of axons terminating instead in the OMPL or in both the OMPL and the IPL (Figure S5g–i). Despite this change in axon position, mutant OFF-CBCs showed typical bipolar morphologies and extended dendrites that terminated properly in the OPL, as in the WT retina (Figure S5g–i). Thus, unlike its role in ACs, FAT3 is not essential for the position or shape of OFF-CBC dendritic arbors.

**Figure 6:**
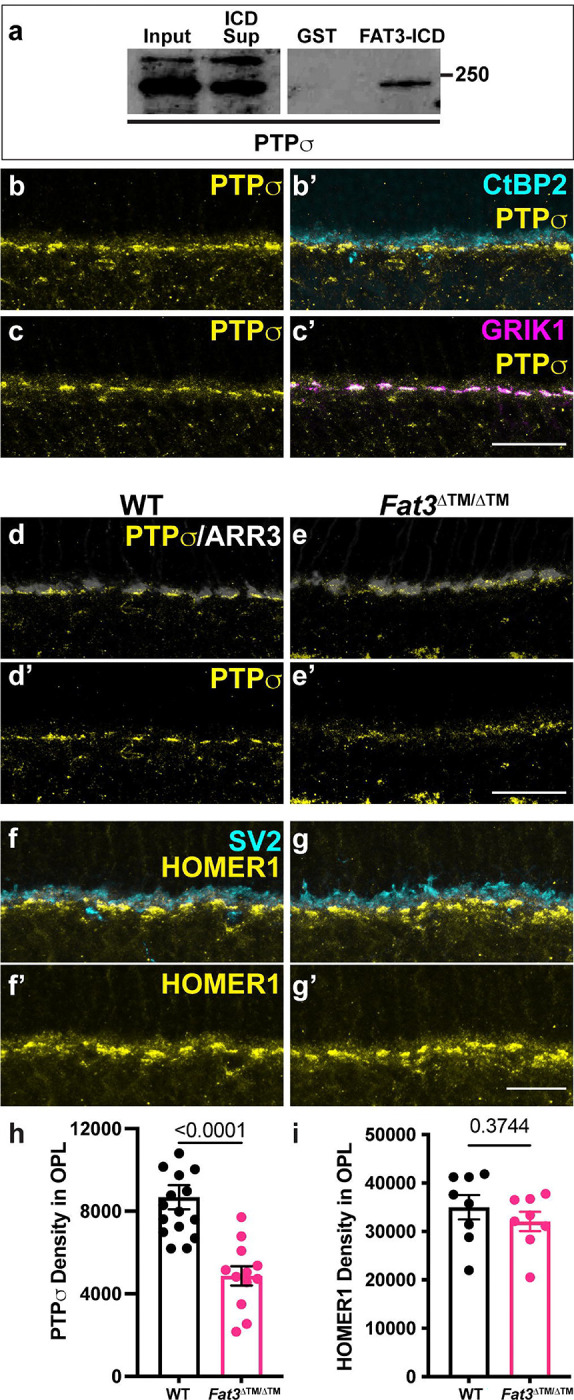
Assay of FAT3 binding to PTPσ and PTPσ localization in WT and FAT3-ICD mutant. (a) Binding of FAT3-ICD to PTPσ was assayed using a pull down, with GST fused to FAT3-ICD. (b) Immunostaining of PTPσ and (b’) CtBP2, a marker of ribbons in photoreceptor axons. (c) Immunostaining of PTPσ and (c’) GRIK1. (d) Immunostaining of PTPσ in WT retinas. (e) Immunostaining of PTPσ in *Fat3*^ΔTM/ΔTM^ retinas. (f) Immunostaining of HOMER1 and SV2, and (f’) HOMER1 alone in WT retinas. (g) Immunostaining of HOMER1 and SV2, and (f’) HOMER1 alone in *Fat3*^ΔTM/ΔTM^ retinas. (h) Quantification of PTPσ integrated intensity in the OPL. WT Controls: 8682 ± 583 (n=16 sections, N=4 animals); *Fat3*^ΔTM/ΔTM^: 4871 ± 463.5 (n=12 sections, N=3 animals), Mann-Whitney test. (i) Quantification of HOMER1 integrated intensity in the OPL. WT Controls: 34997 ± 2509 (n=8 sections, N=3 animals); *Fat3*^ΔTM/ΔTM^: 32051 ± 2003 (n=8 sections, N=3 animals), t test. Scale bars: 20μm.

**Figure 7:**
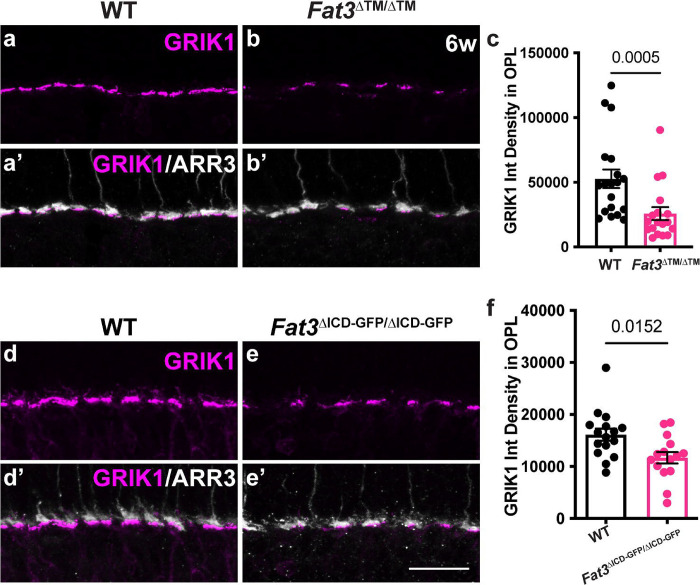
Immunohistochemical assay of GRIK1 in WT and *Fat3*^ΔTM/ΔTM^ retinas. (a) Immunostaining for GRIK1 in WT retinas. (b) Immunostaining for GRIK1 in *Fat3*^ΔTM/ΔTM^ retinas. Cone arrestin (ARR3) labels the cone photoreceptor axonal endings in the OPL in a’ and b’. (c) Quantification of GRIK1 integrated intensity in the OPL. WT Controls: 52727 ± 7204 (n=19 sections, N=4 animals); *Fat3*^ΔTM/ΔTM^: 25838 ± 5028 (n=18 sections, N=4 animals), Mann-Whitney test. (d) Immunostaining for GRIK1 in WT retinas. (e) Immunostaining for GRIK1 in *Fat3*^ΔICD-GFP/ΔICD-GFP^ retinas. Cone arrestin (ARR3) labels the cone photoreceptor endings in the OPL in d’ and e’. (f) Quantification of GRIK1 integrated intensity in the OPL. WT Controls: 16119 ± 1168 (n=16 sections, N=4 animals); *Fat3*^ΔICD/ΔICD^: 11687 ± 1110 (n=15 sections, N=4 animals), Mann-Whitney test.

Given the absence of obvious changes in position or morphology of BC dendrites, we hypothesized that the observed changes in retinal function are due instead to FAT3-dependent effects on retinal ribbon synapses. Indeed, in addition to binding to a variety of cytoplasmic effectors important for neuronal migration and neurite retraction, the FAT3 ICD binds to several proteins associated with synaptic function, such as HOMER1^6^ and the LAR family protein, PTPσ, which is encoded by the *Ptprs* gene, as shown by GST-pulldown (Figure 6a). Further, *Drosophila* Fat-like interacts with the related RPTP protein dLAR^27^, and PTPσ is required for excitatory synapse formation in hippocampal neurons^28–30^. Since *Ptprs* RNA is detected in both ON- and OFF-BCs^2^ (Figure S2), we asked whether cell type-specific synaptic features might be altered in the absence of FAT3. Immunostaining revealed that PTPσ localized to the post-synaptic dendrites of CBCs, co-localized with GRIK1 and apposed CtBP2+ ribbons in the cones (Figure 6b,c). However, significantly less PTPσ was detected in the OPL of *Fat3*^ΔTM/ΔTM^ mutants (density in the OPL of 4871 ± 463.5, n=3 mutants vs 8682 ± 583.0 in n=4 WT) (Figure 6d,e,h). Immunostaining revealed no change in the levels of pre-synaptic CTBP2+ ribbons (OPL mean fluorescence intensity: 5.00 ± 0.14 in n= 4 *Fat3*^ΔTM/ΔTM^ animals *vs.* 4.80 ± 0.16 in n=4 WT; 9.70 ± 0.39 in n= 4 *Fat3*^ΔICD-GFP/ΔICD-GFP^ animals *vs.* 10.03 ± 0.32 in n=4 WT) (Figure S6a,b,e,g,h,k) or post-synaptic HOMER1 in the OPL of *Fat3* mutants (Figure 6f,g,i), indicating that FAT3 is not required for synapse formation *per se*. Moreover, GRIK1 staining was severely reduced in the OPL of both *Fat3*^ΔTM/ΔTM^ (Figure 7a–c) and *Fat3*^ΔICD-GFP/ΔICD-GFP^ (Figure 7d–f) retinas compared to WT littermates (density in the OPL: 25838 ± 5028, n=4 *Fat3*^ΔTM/ΔTM^ vs 52727 ± 7204 in n=4 WT and 11687 ± 1110 in n= 4 *Fat3*^ΔICD-GFP/ΔICD-GFP^ *vs.* 16119 ± 1168 in n=4 WT). BCs also expressed less *Grik1* RNA in *Fat3*^ΔTM/ΔTM^ animals compared to WT (Figure S7a–c). Loss of GRIK1 was apparent by 3 weeks of age in *Fat3*^ΔTM/ΔTM^ mice, when 30 Hz flicker ERG defects were already present (Figure S7e–k). By contrast, we did not detect a significant change in the expression or localization of the ON-CBC synaptic protein GRM6, with similar levels of *Grm6* RNA in the INL of mutant and control retinas (Figure S7a,b,d) and of GRM6 in the OPL of mutant and control retinas (Figure S6c,d,f). GRM6 levels were also unchanged in *Fat3*^ΔICD-GFP/ΔICD-GFP^ mutants (Figure S6i,j,l). Loss of *Ptprs* (Figure S8a,b) also resulted in a modest decrease of GRIK1 in the OPL (Figure S8c,d,g) without significantly affecting the ribbons of the photoreceptors (Figure S8e,f,h). However, unlike *Fat3* mutants, *Ptprs*^−/−^ animals did not show any change in the ERG response to a 30 Hz flickering stimulus, the implicit timing of the response to a 20 Hz stimulus, or the d-wave amplitude in the step ERG assay (Figure S8i–k). Thus, FAT3 likely impacts not only the presence of PTPσ and GRIK1, but also additional features of the CBC synapses necessary for responses to high temporal frequency stimuli.

## Discussion

A major goal in neuroscience to understand how the diverse cell types of the retina, including >80 types of interneurons, influence parallel circuits that transform information about specific elements of the visual world. A valuable approach is to characterize retinal physiology and visual behavior in mice carrying mutations in genes required for proper circuit assembly and/or function. However, the nature of the circuits that enable responses to stimuli that change with high temporal frequency has been explored very little, in part because standard assays of retinal function, particularly the conventional scotopic and photopic ERGs, are not designed to detect signals elicited by these stimuli. Despite lacking the cellular level resolution that can be achieved by *ex vivo* patch clamping, the ERG is an elegant and relatively simple measurement that can be used *in vivo* to reveal behaviorally-relevant changes in overall retinal function from population of cells. Here, we use flicker and step ERGs to show that retinal activity in response to high frequency flickering and light-off stimuli is altered in *Fat3* mutant mice, which also show an impaired behavioral response to flickering stimuli. FAT3 is a multi-functional transmembrane protein that is expressed by ACs, BCs, and RGCs and is required for retinal lamination. By analyzing the consequences of *Fat3* deletion from different retinal cell types, we discovered that the loss of high frequency light response was not due to changes in AC position or connectivity. Instead, these visual defects appear to reflect abnormal synaptic responses in BCs. Although the precise origin of the synaptic defects remains unclear, we found that FAT3 binds to the synaptic protein PTPσ and that both FAT3 and PTPσ are required for enrichment of GRIK1 at the cone to OFF-CBC synapse. Further, only the loss of glutamatergic signaling through both ON and OFF CBCs recapitulates the *Fat3* visual deficits. Together, these data demonstrate the importance of both ON and OFF CBCs for high temporal frequency light response, and uncover a role for FAT3 in the formation and/or maintenance of functional cone to BC synapses.

### FAT3 mutant mice show deficits in high frequency light response

Despite their severe defects in neuronal wiring, *Fat3* mutant mice are not blind, as seen by the preservation of conventional ERG and optomotor responses. However, when presented with fast flickering stimuli, *Fat3* mice showed severe visual deficits consistent with loss of signaling from cones to OFF-CBCs. In support of this idea, abnormal high temporal frequency light response was observed only in mice lacking *Fat3* in BCs and did not correlate with effects on retinal lamination (Figure 2,3,4). Additionally, *Fat3* is expressed by multiple BC types, including GRIK1-positive OFF-CBCs, and GRIK1 levels were reduced in the OPL of *Fat3* mutant mice (Figure 1,7). To independently assay cone to OFF-CBC signaling, we established a step ERG protocol and showed that the d-wave was reduced in both *Fat3* and *Grik1*^−/−^ mutant mice compared to WT littermates (Figure 2,4,5). Collectively, these data suggest that FAT3 is required for proper OFF-CBC function and hence the ability to detect fast flickering stimuli. This phenotype is fundamentally different from that which occurs in *Fat3* mutant ACs, which extend extra neurites that form ectopic synapses.

### Both ON and OFF BCs are required for high frequency light response

BCs are the first retinal interneurons that encode, segregate, and relay visual information into over a dozen pathways for further processing. It had been hypothesized that the OFF-CBCs mediate high frequency visual signal transmission, based upon indirect evidence from flicker ERG studies of mice carrying mutations such as *Grm6*^−/− 11^. Here, we found that both ON and OFF CBCs are required for high frequency light response (Figure 5). Inspired by the loss of GRIK1 from *Fat3* mutant OFF-CBC synapses, we assayed visual responses in mutant mice lacking critical glutamate receptors specific to either ON (GRM6) or OFF (GRIK1) CBCs. However, in contrast to the shared effects on the d-wave, *Grik1*^−/−^ mice showed only a modest reduction in the amplitude of the response to a 30 Hz flicker ERG. This was striking given the drastic reductions in this measurement in *Fat3* mutants. These findings raised the possibility that FAT3 is also required for ON-CBCs to transmit high frequency signals. Indeed, mice lacking both *Grm6* and *Grik1* showed a similar reduction in high frequency light detection as *Fat3* mutants. Other physiological phenotypes also were seen: *Grik1*^−/−^ mice had advanced implicit time in response to 20 Hz, while *Grm6*^−/−^ mice had delayed implicit time, similar to *Fat3* mutants. These data strongly suggest a role for both ON and OFF classes of CBCs in supporting high frequency visual signal transmission.

OFF-CBC activities are thought to be initiated by two classes of ionotropic GluRs, namely kainate and AMPA receptors, which were proposed to differentially encode temporal signals from cones^31^. AMPA receptors also have been reported to mediate high frequency signaling in cb2, a particular OFF-CBC subtype in ground squirrel retina, as shown by *ex vivo* patch recording^32^. Here, we found that *Grik1*^−/−^;*Grm6*^−/−^ mice lacked all responses to 30 Hz flicker ERGs, arguing against a role for AMPA receptors in the bulk transmission of high temporal frequency stimuli from cone to CBCs in mice, at least not at the population level that is detectable by *in vivo* ERG. Despite some controversy on this topic in mice^33,34^, AMPA receptors might mediate only slow and/or sustained OFF-CBCs responses that do not contribute to the transient and/or fast responses needed for high temporal frequency visual signal transmission, as suggested by a previous *ex vivo* study using pharmacological blockers with patch recordings^35^.

How FAT3 impacts ON-CBC function remains unclear. Although *Fat3* RNA is weakly expressed in the 5D and 6 ON-CBC subtypes^2^ (Figure S2), possibly affecting some ON-CBC functions directly, it is unlikely that an autonomous change in only two subtypes would alter the ON-CBC population responses that the ERG measures. Further, *Fat3* mutants showed no detectable change in the level of GRM6, the glutamate receptor that leads to membrane voltage changes in ON bipolar cells^24^. OFF-CBCs are connected to ON-BCs via AII amacrine cells in IPL. However, this circuit is unlikely to affect the flicker ERG, which originates from the cation channels of BCs in or near the OPL^9,36^. However, it is possible that modulation originating within the IPL affects OPL signaling through an undescribed backpropagation mechanism, which was suggested by an *ex vivo* study using pharmacological blockers within the amphibian retina^37^. We propose three possible explanations for the observations of the role of the ON-CBC: 1) GRM6 protein levels in ON-CBC are affected in *Fat3* mutants but we are unable to resolve them, as there is abundant GRM6 in RBCs, the most numerous of all BCs, 2) FAT3 regulates certain GRM6 functions and/or related signaling pathways (e.g. G proteins or TRPM1, which would also be masked by their expression in RBCs) that we did not assay, and/or 3) GRM6 is not directly downstream of FAT3 but can act in concert with FAT3-dependent signaling in OFF-CBCs and/or ACs to modulate high frequency visual signal transmission. For example, PTPσ might serve as an intermediary, as it can act in *trans*, is expressed in both ON and OFF-BCs^2^ (Figure S2), and is reduced in *Fat3* mutants (Figure 6). Further studies, including detailed patch clamp analysis, are needed to address these possible explanations.

It is also interesting that the d-wave was completely gone in *Grik1*^−/−^;*Grm6*^−/−^ eyes, but only slightly reduced in *Grik1*^−/−^ eyes, again suggesting an unexpected role for ON-BCs in the response to light turning off. One possible explanation concerns the phenomenon of lateral inhibition. An individual ON-BC can be depolarized instead of hyperpolarized when there is no light in its peripheral receptive field, i.e. surrounding the central receptive field^38^. ON-BCs were originally termed “ON-center” BCs to accurately reflect their response to light in their central and peripheral fields. Since the ERG light stimulus is full-field, it is possible that the effect of having the light turn off in the peripheral receptive field of individual GRM6+ ON bipolar cells contributes to the residual d-wave in *Grik1*^−/−^ retina.

### *Fat3* effects on high temporal frequency light response requires intracellular signaling

The discovery of a new synaptic defect in *Fat3* mutant retinas highlights FAT3’s versatility as a signaling molecule. Previous work showed that FAT3 acts through different motifs in its ICD to control AC migration, neurite retraction, and synapse localization, likely by recruiting different combinations of cytoplasmic effectors^6^. Here, we found that the FAT3 ICD is also required for CBC function, possibly acting through a separate module of synapse-related effectors. The FAT3-ICD interacts with several known synaptic proteins, including PTPσ, which is one of the four type IIA family of receptor-type protein tyrosine phosphatase in the LAR-RPTP subfamily^39^. Interactions between FAT3 and PTPσ appear to regulate the amount of GRIK1 at the synapse, since PTPσ levels were reduced in the OPL of *Fat3* mutants (Figure 6) and GRIK1 levels were reduced in the OPL of both *Fat3* and *Ptprs* mutants (Figure S8). Although PTPσ plays a well-established role in differentiation of the pre-synaptic component of excitatory synapses in the brain^28,30,39^, our findings point to a role on the post-synaptic side, as suggested previously^29^. Further, the related protein LAR also can be localized to the post-synaptic compartment and is required for proper surface expression and clustering of AMPA receptors^40^. Thus, it is possible that PTPσ acts similarly to control the distribution of GRIK1 in CBCs, either on its own or in collaboration with FAT3. Since *Ptprs* mutants do not show the same visual deficits as *Fat3* mutants (Figure S8), other LAR subfamily members may compensate. This might also explain why *Ptprs* mutants have no obvious changes in retinal lamination^41^. Alternatively, other FAT3-dependent proteins may enable sufficient GRIK1 activity for OFF-CBC signaling in *Ptprs* mutants. Although much remains to be learned about the contribution of FAT3-PTPσ interactions to the synapse, this seems to be a conserved relationship, since *Drosophila* Fat-like and LAR interact to ensure collective cell migration^27^, in this case acting in *trans*^42^.

There are several ways that FAT3 might influence synaptic function. One model is that the FAT3 ICD serves as a scaffold for synaptic proteins that secures them to the OFF-CBC dendrites in the OPL and thus directly shapes visual signal transmission. This fits with the fact that FAT3, PTPσ, and GRIK1 are all localized to the OPL, and that several other synaptic proteins, including the WAVE regulatory complex, also interact with the FAT3 ICD^6^. Alternatively, FAT3 may ensure directed trafficking of PTPσ and other proteins to the synapse, echoing its role as a tissue polarity protein and its ability to promote asymmetric localization of cytoskeletal proteins^8^. These possibilities are not mutually exclusive, as FAT3 could control synapse assembly during development and then maintain the synapse in the mature retina. Finally, FAT3 may impact the synapse through effects on gene expression. In flies, the Fat-like ICD is cleaved and binds to a transcriptional co-repressor to influence gene expression^43^. Since the LAR ICD can be internalized and thus inhibit transcription^44^, FAT3 and PTPσ could cooperate to control expression of synaptic genes. This notion is supported by the observation that *Grik1* RNA is modestly reduced in *Fat3* mutant bipolar cells (Figure S6).

#### Physiological consequences of high frequency light response

Finally, it is interesting to mention some recent studies reporting a benefit of gamma frequency entrainment. A one-hour daily exposure of 40 Hz light flashes plus 40 Hz sound was reported to alleviate dementia symptoms and neurodegeneration in mouse models of Alzheimer’s disease, and in human patients^45–47^. The data shown here concerning how high temporal frequency light signals are transmitted at the first synapse may contribute to our understanding of the mechanisms of gamma frequency entrainment. These data may also help resolve the different results reported in a recent study of a mouse model of Alzheimer’s disease^48^ where 40 Hz light stimulation provided little benefit.

### Limitations of the study

Although our data provide strong evidence that FAT3 impacts cone-CBC signaling needed for high frequency light response, this study does not show definitively what is wrong at the level of the synapse. The ERG is a measurement of cellular activity across a population of cells, i.e. a group of cells from the same class that function similarly, such as OFF-CBC vs. ON-CBC. Due to the lack of Cre lines specific for all BCs, or OFF-CBCs, or ON-CBCs, it is not possible to analyze the consequences of FAT3 loss in these cell types. Additionally, despite being an excellent and relatively easy tool to assay overall retinal physiology of cell populations *in vivo*, the ERG cannot distinguish electrical activity limited to a particular subtype, e.g. CBC-5D. ERGs are also not designed to detect signals carried within distinct microcircuits that perform transformations of particular features of a visual scene. Patch clamping is necessary to study the responses of specific BC types at single-cell resolution. Likewise, the large size of FAT3 and its localization to the cell surface make it difficult to define the precise nature of its effects on PTPσ, GRIK1 and other uncharacterized players. Our discovery that CBC activity is compromised in *Fat3* mutants sets the stage for more detailed analysis in the future.

## Methods

### Animals

The *Fat3*^ΔTM^ mouse line lacks exon 23, which contains the coding region for the transmembrane domain. Since no ICD anchored to the membrane has been detected^6,7^, this allele is expected to act as a full loss of function. The *Fat3*^floxed^ line contains LoxP sites flanking exon 23^7^. The *Fat3*^ΔICD-GFP^ mouse line has a deletion of most of the FAT3-ICD, which is replaced by GFP. This line possesses a full extracellular domain anchored to the cell membrane^6^. *Fezf2*^−/−^ mice were generated by Hirata and colleagues^49^. Heterozygous mice were used as breeders to obtain wildtype and knockout littermates. *Grik1*^−/−^ mice were obtained from Christophe Mulle (University of Bordeaux, France)^50^. *Grm6*^−/−^ mice (also known as *Grm6*^*nob3*^), which was characterized by Maddox and colleagues^51^, were purchased from The Jackson Laboratory (ME, Strain #: 016883). *Grik1*^−/−^;*Grm6*^−/−^ mice were bred in house by crossing the two lines. *Ptprs* KO mice were made by Michel Tremblay’s laboratory (McGill University)^52^. Transgenic mice expressing Cre recombinase were obtained from the following sources: *Ptf1a*^CRE^ (C. Wright, Vanderbilt U.)^19^; *Islet1*^CRE^ (a.k.a. *Isl1*^*tm1(cre)Sev*^/J)^53^ (The Jackson Laboratory, ME. Strain #: 024242); and *Bhlhe22*^CRE^ (a.k.a *Bhlhb5*^CRE^)^54^ (M.E. Greenberg, Harvard Medical School). Mice were maintained on a 12 hour/12 hour light/dark cycle at 18–23 °C and 40–60% humidity. Animals were handled ethically according to protocols approved by the Institutional Animal Care and Use Committee at Harvard Medical School. Genotyping was done using real time PCR (Transnetyx, Cordova, TN).

### Electroretinography (ERG)

Mice were dark adapted overnight before *in vivo* ERG recordings. Animals were anesthetized with 100/10 mg/kg ketamine/xylazine cocktail and placed on a heating pad. Their pupils were dilated with a drop of 1% tropicamide solution (Bausch + Lomb). Electrodes were applied to the cornea to pick up the electrical signals from the retina. Eyes were kept moist by a drop of phosphate buffered saline (PBS). With an Espion E3 System (Diagonsys LLC), four types of ERG tests were performed: 1) scotopic test; 2) photopic tests with 1, 10, 100 and 1,000 cd s/m^2^ under a 30 cd/m^2^ background light to saturate the rod responses; 3) flicker tests at 0.5, 10, 20, 30, 40 and 50 Hz; and 4) step-light test with a three-second light step of 1,000 cd/m^2^. The scotopic and photopic ERGs were conducted as described previously^55^. The flicker ERG was recorded using 3.162 cd s/m^2^ flashes as adapted from a published protocol^11^. The step ERG was created for this study to probe the d-wave from OFF-bipolar cells.

### Optical Coherence Tomography (OCT)

OCT of mouse eyes was conducted using an OCT2 system (Phoenix Research Labs), as described previously^55^. OCT imaging was performed on the mice *in vivo* immediately after the ERG tests to confirm the previously observed *ex vivo* histological changes. Before OCT imaging, a drop of GONAK 2.5% hypromellose solution (Akorn) was applied to the eye as the immersion medium with the OCT lens.

### Flicker-light cued fear conditioning assay

The fear conditioning test for high temporal frequency vision was created by modifying previously published protocols^16^. The Med Associates (St Albans, VT) system was used for the tests with four LED lights (two green and two yellow) controlled by a computer software (Med PC). Mice were videotaped through Media Recorder Software and the fear response of freezing was analyzed by researchers who were blinded to the genotypes as a surrogate measurement of memory linked with visual input. On Day 1, the mice were brought to the electric shock cage individually to get familiar with the environment and procedure. They were in the cage for 30 minutes under a dim house light in the background and LEDs turned off (Figure S2). On Day 2, mice were brought back to the electric shock cage, first exposed to static green/yellow LED lights for two minutes, followed by 30 seconds of 33 Hz LED flicker light (i.e. the cue). Within the last 2 seconds of the cue, a series of 0.7mA shocks were initiated to trigger the fear memory linked with the cue. This static-flicker-shock cycle was repeated twice more. On Day 3, contextual memory (i.e. context test) was measured by placing the mouse back into the conditioning chamber for three minutes (no electric shock was delivered during this session), and the duration of freezing was recorded. Then, cued memory was measured by placing the mouse into an altered context, which was composed of different tactile and olfactory cues. The amount of freezing in the altered context was measured as a baseline (3 minutes, static light) followed by measurement of freezing during presentation of the cued stimulus (3 minutes, 33 Hz flicker light). The videos were analyzed by an examiner blinded to the genotypes to extract the freezing time of all three conditions (i.e. context, static and 33 Hz flicker).

### Optomotor assay

Using an OptoMotry System (CerebralMechanics), the optomotor assay to measure the visual acuity of mice was conducted as described previously^55^. The testing grates were set with 100% contrast and were moved at 1.5 Hz temporal frequency. The visual acuity (i.e. maximal spatial frequency in the unit of cycle/degree) was tested by an examiner, who was blinded to the genotype. During each testing episode, the examiner reported either “yes” or “no” to a computer program until the threshold of acuity was reached. The parameter of each testing episode (i.e. spatial frequency) was determined by the computer program and blinded to the examiner.

### Dissections and immunohistochemistry

Animals of the desired postnatal age (3 or 6 weeks of age, as indicated) were euthanized by CO_2_ inhalation and cervical dislocation. Extraocular tissue, the cornea and the lens were removed from the eyes and the eyecups were further fixed by immersion in 4 % paraformaldehyde (PFA, EMS Cat#15710) for 30 minutes (min) at room temperature or 15 min on ice. After several washes with PBS buffer, the eyes were submerged in 30% sucrose and kept at 4°C for at least 2 hours (h). After sucrose cryoprotection, eyes were incubated in NEG-50 (VWR, Cat#84000-154) overnight at 4°C and embedded by freezing in a liquid nitrogen vapor bath. Retinal slices were obtained by cryosectioning the eyes at 20 μm thickness and mounting on Superfrost^®^ Plus Micro Slide (VWR, Cat#48311-703). The sections were either stained immediately or stored at −80°C.

For regular immunohistochemistry, NEG-50 was removed by short incubation in PBS and then sections were blocked and permeabilized by incubation in 5% Normal Donkey Serum (NDS, Jackson ImmunoResearch Cat#017-000-121) in Sorensońs supplemented with 0.5% Triton-X for 1–2 h at room temperature. Sections were then incubated in primary antibody diluted in blocking buffer overnight at 4°C. After several washes with PBS, sections were incubated with fluorescent secondary antibodies diluted in 5% NDS in Sorensońs buffer supplemented with 0.02% Triton-X for 1.5–2 h at room temperature. After final washes, sections were mounted in DAPI-Fluoromount-G (SouthernBiotech Cat#0100–20). Primary antibodies used for immunohistochemistry were: rabbit anti-ARR3 (Millipore Sigma, Cat#AB15282), goat anti-Bhlhb5 (1:500; Santa Cruz Biotechnology, Cat#sc-6045), mouse anti-CtBP2 (1:2,000, BD Biosciences, Cat#612044), rabbit anti-dsRed (cross-reacts with TdTomato, 1:1,000, Clontech, Cat#632496), mouse anti-FAT3^6,7^ (1:200), chicken anti-GFP (1:500; Aves, Cat#GFP-1020), mouse anti-GRIK1 (GluR5, 1:200, Santa Cruz Biotechnology, Cat#sc-393420)^25^, sheep anti-GRM6 (1:2,000, a gift from Jeannie Chen, USC and originally developed by Kirill Martemyanov Lab^56^), goat anti-PTPσ (1:200; R&D Systems, Cat# AF3430), rabbit anti-VGAT (1:300; SynapticSystems, Cat#131002), and mouse anti-VSX2 (Chx10, 1:100; Santa Cruz Biotechnology, Cat#sc-365519). All secondary antibodies were diluted 1:1,000 and were: Donkey anti-chicken Alexa Fluor^®^ 488, Donkey anti-goat Alexa Fluor^®^ 568, Donkey anti-mouse Alexa Fluor^®^ 488, Donkey anti-mouse Alexa Fluor^®^ 568, Goat anti-mouse Alexa Fluor^®^ 647, Donkey anti-rabbit Alexa Fluor^®^ 488, Donkey anti-rabbit Alexa Fluor^®^ 568, Donkey anti-rabbit Alexa Fluor^®^ 647, and Donkey anti-sheep Alexa Fluor^®^ 568.

To detect FAT3 on retinal sections, we performed Hybridization Chain Reaction Immunohistochemistry (HCR-IHC)^57^, according to the manufacturer’s instructions (Molecular Instruments, CA). In brief, on day 1 sections were treated similarly to regular immunohistochemistry. After overnight incubation of the primary mouse anti-FAT3^7^ (1:200) antibody, sections were rinsed with PBS-0.1% Tween-20 (PBS-T) and incubated with 1 μg/mL of initiator-labeled anti-mouse secondary antibody (Molecular Instruments) for 1 h at room temperature. Slides were rinsed with PBS-T and a final rinse with 5X Saline-Sodium Citrate buffer with 0.1% Tween-20 (SSC-T) and incubated with amplification buffer (Molecular Instruments) for 30 min at room temperature. H1 and h2 fluorescently-labeled hairpins were separately denaturated at 95 °C for 90 s followed by 30 min incubation at room temperature in the dark. A 60 mM hairpin solution mix was prepared by adding snap-cooled h1 and h2 hairpins to amplification buffer and incubated on the slides over night at room temperature in a dark, humidified chamber. After several washes with SSC-T, sections were mounted with DAPI-Fluoromount-G.

### *In situ* hybridization (RNAscope)

Tissue collection was performed similar as to for immunohistochemistry, except using RNAase-free conditions. For RNAscope *in situ* hybridization, we used RNAscope^®^ Fluorescent Multiplex Reagent Kit v2 (ACD, Cat#323120) assay following the manufactureŕs instructions. In brief, retinal sections were post-fixed in 4% PFA for 15 min at room temperature, treated with hydrogen peroxide for 10 min at room temperature and treated with Protease III for 10 min at 40°C before probe incubation. Probes were obtained from ACD (see Key Resource table). Immunohistochemistry was performed after *in situ* hybridization by rinsing the sections in PBS after the final RNAscope wash and permeabilized and blocked again with 5% NDS/0.5% Triton X-100 Sorensońs buffer, followed by the regular immunohistochemistry protocol.

### *In vivo* viral injection

The AAV-Grik1-GFP plasmid was generated by cloning a previously identified Grik1 enhancer (CRM4)^15^ upstream of a simian virus 40 (SV40) intron, Kozak sequence, GFP coding sequence, woodchuck hepatitis virus post-transcriptional regulatory element (WPRE), and polyadenylation sequence. To produce the AAV8-Grik1-GFP vector, HEK293T cells were triple transfected with a mixture of AAV-Grik1-GFP plasmid, adenovirus helper plasmid, and rep2/cap8 packaging plasmid. Viral particles were harvested from the supernatant 72 hours after transfection and purified using an iodixanol gradient as described previously^58^. The titer of AAV8-Grik1-GFP was determined by comparing SYPRO Ruby (Molecular Probes) staining for viral capsid proteins (VP1, VP2, and VP3) to that of a reference vector with known titer.

To deliver AAV8-Grik1-GFP into the developing retina, we injected into the subretinal space as described previously^59,60^. In brief, neonatal P2–3 mouse pups were anesthetized by chilling on ice. We injected 2.5 × 10^9^ vector genomes (vg) per eye, which is at a titer not toxic to the eye, diluted in PBS and 0.1% Fast Green (for visualization) using a pulled borosilicate glass needle with an opening of 0.5–1mm diameter connected to an Eppendorf FemtoJet injector into the subretinal space. The pups recovered on a warm pad and upon regaining consciousness they were returned to their mother. We then let them develop until performing histological procedures at P22.

### Image acquisition

After immunohistochemistry or RNAscope, retinal sections were imaged within 300 μm from the optic nerve head on a Leica SP8 or a Zeiss LSM800 confocal microscope. The entire sections were imaged in consecutive z-slices separated by 1 μm using a 40x or 63x oil objective. The z stacks were then projected at maximum fluorescence intensity using Fiji/ImageJ.

### Histochemical quantifications

We assigned random numbers to each image to ensure blinded quantifications. Only after the quantification was done, the identity of the images was revealed to assign the values to their corresponding genotype. All the procedures were done under the same technical parameters, and the comparisons were made between control and experimental conditions within the same experiment to avoid batch effects. The animal (N) and sample (i.e. sections, n) numbers, statistical test performed, and *p* values are indicated in figure legends and/or figures.

We assessed AC migration and the “ectopic synapse score” or “OMPL score’ as described previously^6^. To quantify expression of PTPσ, GRIK1 or GRM6 proteins in the OPL, the images were thresholded until background signal in the OPL was not observed. All images from the same experiment were treated the same way using Fiji (ImageJ). Then, the integrated density was measured to quantify protein expression in the same total area of each image on the OPL region (44.69 μm × 17.78 μm). To quantify CtBP2 fluorescence intensity on immunohistochemistry samples we used Fiji (ImageJ) to measure the Mean Gray Value on areas of the OPL by tracing a rectangle that took up most of the OPL height. In addition, the Mean Gray Value was measured on a rectangle traced on the ONL (region where photoreceptors reside and is used as background signal) to normalize the value of the OPL.

### Statistics

To determine significant differences between control and experimental groups, we used Prism6 software for statistical analysis. After applying a D’Agostino-Pearson omnibus normality test to determine Gaussian distribution of the samples, we either used two-tailed *t* test (if the samples followed a Gaussian distribution) or Mann-Whitney test (if the samples did not follow a Gaussian distribution) to calculate the *p* values. For ERG data of more than two groups, one-way ANOVA with Dunnett multiple comparison test was used.

### GST Pull down and Western blot

For binding analysis, we performed Western blots of supernatants after pulling down binding partners from mouse brain protein homogenates with the FAT3-ICD fused to Glutathione-S-transferase (GST) and GST alone generated previously^6^. Samples were denatured at 95°C for 10 mins and subjected to SDS-PAGE in a 4–12% Criterion^™^ XT Bis-Tris Protein Gel (Bio-Rad) using XT MES Running Buffer (Bio-Rad). After 2 h at 150 V of electrophoresis, the proteins were transferred to Immobilon-P PVSF (0.45μm, Sigma-Millipore) in Tris-Glycine buffer supplemented with 20% methanol for 1 h at 75V. The Immobilon-P membranes were blocked with 5% skim milk in TBS buffer and then incubated with primary antibodies at 4°C overnight. The primary antibody used for Western blots was mouse anti-PTPσ (1:1,000, Medimabs, Cat# MM-0020-P). After several washes with TBS supplemented with 0.5% Tween 20 (Sigma-Aldrich), the membranes were incubated with a secondary goat anti-mouse HRP antibody (Biorad, Cat# 170-6516) diluted 1:2,000 for 1–2 h at room temperature. The signal was developed using Clarity ECL substrate following the manufactureŕs instructions (Bio-Rad). Western blots were done at least twice with similar results.

### External gene expression profile datasets

Gene expression in different types of retinal bipolar cells was analyzed by using the single-cell RNAseq database and a modified R script that were published previously^2^.

### Resource and data availability

The source data for graphs are provided with this paper. For all quantifications, the raw data are shown along with means and standard errors of the means as well as the statistical analyses utilized. The original images used to generate these data are available from the corresponding author upon request. All unique materials generated in this study are available upon request.

## Supplementary Material

Supplement 1

## Figures and Tables

**KEY RESOURCES TABLE:** 

Reagent or resource	Source	Identifier
**Antibodies**		
Rabbit anti-ARR3	Millipore Sigma	Cat#AB15282; RRID:AB_1163387
Goat anti Bhlhb5	Santa Cruz	Cat#sc-6045; RRID:AB_2065343
Mouse anti-CTBP2	BD Biosciences	Cat#612044; RRID:AB_399431
Rabbit anti-dsRed	Clontech	Cat#632496; RRID:AB_10013483
Mouse anti FAT3	^6,7^	N/A; RRID:AB_2904260
Chicken anti GFP	Aves	Cat#GFP-1020; RRID:AB_10000240
Mouse anti-GRIK1 (GluR5)	Santa Cruz Biotechnology	Cat#sc-393420; RRID:AB_2716684
Sheep anti-GRM6	^56^	N/A
Goat anti-PTPσ	R&D Systems	Cat#AF3430; RRID:AB_2175157
Mouse anti-PTPσ	Medimabs	Cat# MM-0020-P
Rabbit anti VGAT	Synaptic systems	Cat#131002; RRID:AB_887871
Mouse anti-VSX2 (Chx10)	Santa Cruz Biotechnology	Cat#sc-365519; RRID:AB_10842442
Donkey anti chicken, Alexa Fluor^®^ 488	Jackson ImmunoResearch	Cat#703-545-155; RRID:AB_2340375
Donkey anti goat, Alexa Fluor^®^ 568	Thermo Fisher Scientific	Cat#A11057; RRID:AB_142581
Donkey anti mouse, Alexa Fluor^®^ 488	Abcam	Cat#ab150105; RRID:AB_2732856
Donkey anti mouse, Alexa Fluor^®^ 568	Thermo Fisher Scientific	Cat#A10037; RRID:AB_2534013
Goat anti mouse, Alexa Fluor^®^ 647	Thermo Fisher Scientific	Cat#A-21235; RRID:AB_2535804
Donkey anti rabbit, Alexa Fluor^®^ 488	Thermo Fisher Scientific	Cat#A21206; RRID:AB_2535792
Donkey anti rabbit, Alexa Fluor^®^ 568	Thermo Fisher Scientific	Cat#A10042; RRID:AB_2534017
Donkey anti rabbit, Alexa Fluor^®^ 647	Thermo Fisher Scientific	Cat#A31573; RRID:AB_2536183
Donkey anti sheep, Alexa Fluor^®^ 568	Thermo Fisher Scientific	Cat#A-21099; RRID:AB_2535753
Goat anti mouse - HRP	BioRad	Cat# 170-6516; RRID:AB_11125547
		
**Commercial assays**		
RNAscope^®^ Multiplex	ACD	Cat#323100
Fluorescent Reagent Kit v2		
HCR IHC Bundle	Molecular Instruments	N/A
		
**Experimental models: Organisms/Strains**		
Mouse: *Ptf1a*^CRE^	C. Wright, Vanderbilt U ^19^	MGI:2387812
Mouse: *Isl1*^CRE^	The Jackson Laboratory^53^	Strain #: 024242
Mouse: *Bhlhe22*^CRE^	M.E. Greenberg (Harvard Medical School)^54^	N/A
Mouse: *Fat3*^floxed^	^7^	N/A
Mouse: *Fat3*^ΔTM^	^7^	N/A
Mouse: *Fat3*^ΔICD-GFP^	^6^	N/A
Mouse: *Fezf2*^−/−^	^49^	N/A
Mouse: *Grikl*^−/−^	Christophe Mulle (University of Bordeaux, France)^50^	N/A
Mouse: *Grm6*^−/−^	The Jackson Laboratory^51^	Strain #: 016883
Mouse: *Ptprs*^−/−^	Michel Tremblay^52^	MGI:2158757
		
***In situ* hybridization Probes**		
RNAscope^®^ Probe-Mm-*Fat3*-O1	ACD	Cat# 509051
RNAscope^®^ Probe-Mm-*Grik1*-C3	ACD	Cat# 438771-C3
		
**Recombinant DNA**		
AAV-Grik1-GFP plasmid	This paper	N/A
		
**Viral vectors**		
AAV8-Grik1-GFP	This paper	N/A
