# Supplementary figures and images for: High temporal frequency light response in mouse retina requires FAT3 signaling in bipolar cells
Source: bioRxiv. 2024 Jun 28:2023.11.02.565326. Originally published 2023 Nov 4. Preprint. [Version 2] doi: 10.1101/2023.11.02.565326 (PMC10635074; doi:10.1101/2023.11.02.565326)

**Figure S1**

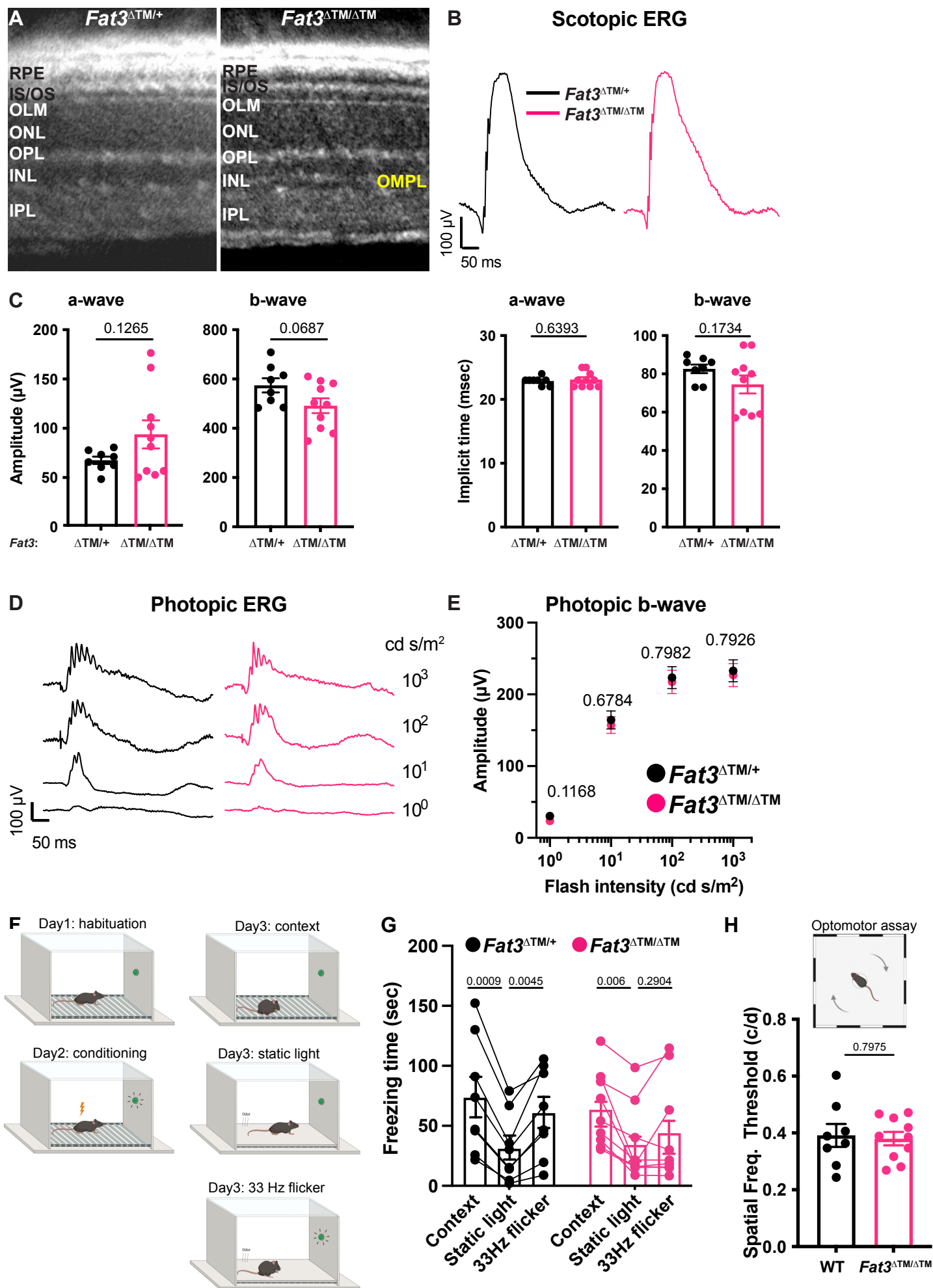

Figure S2

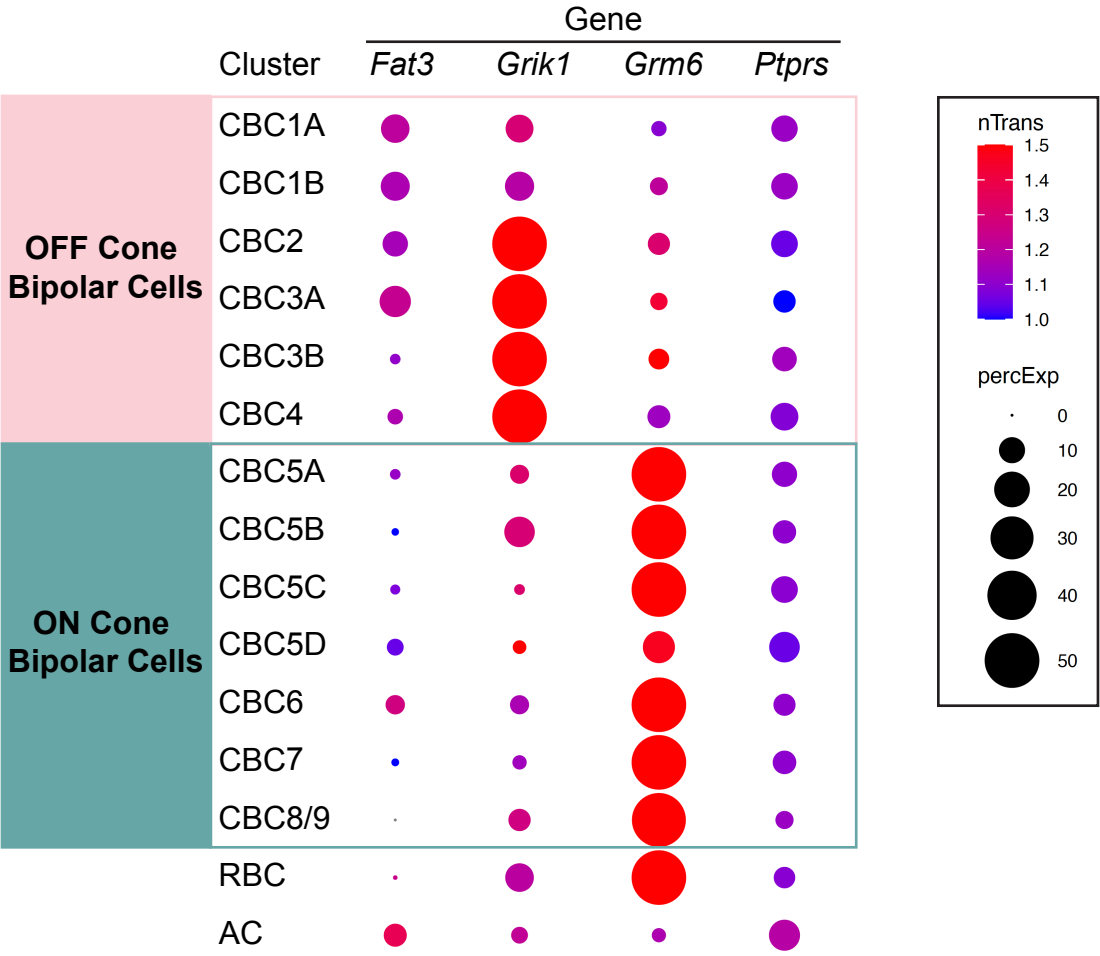

**Figure S3**

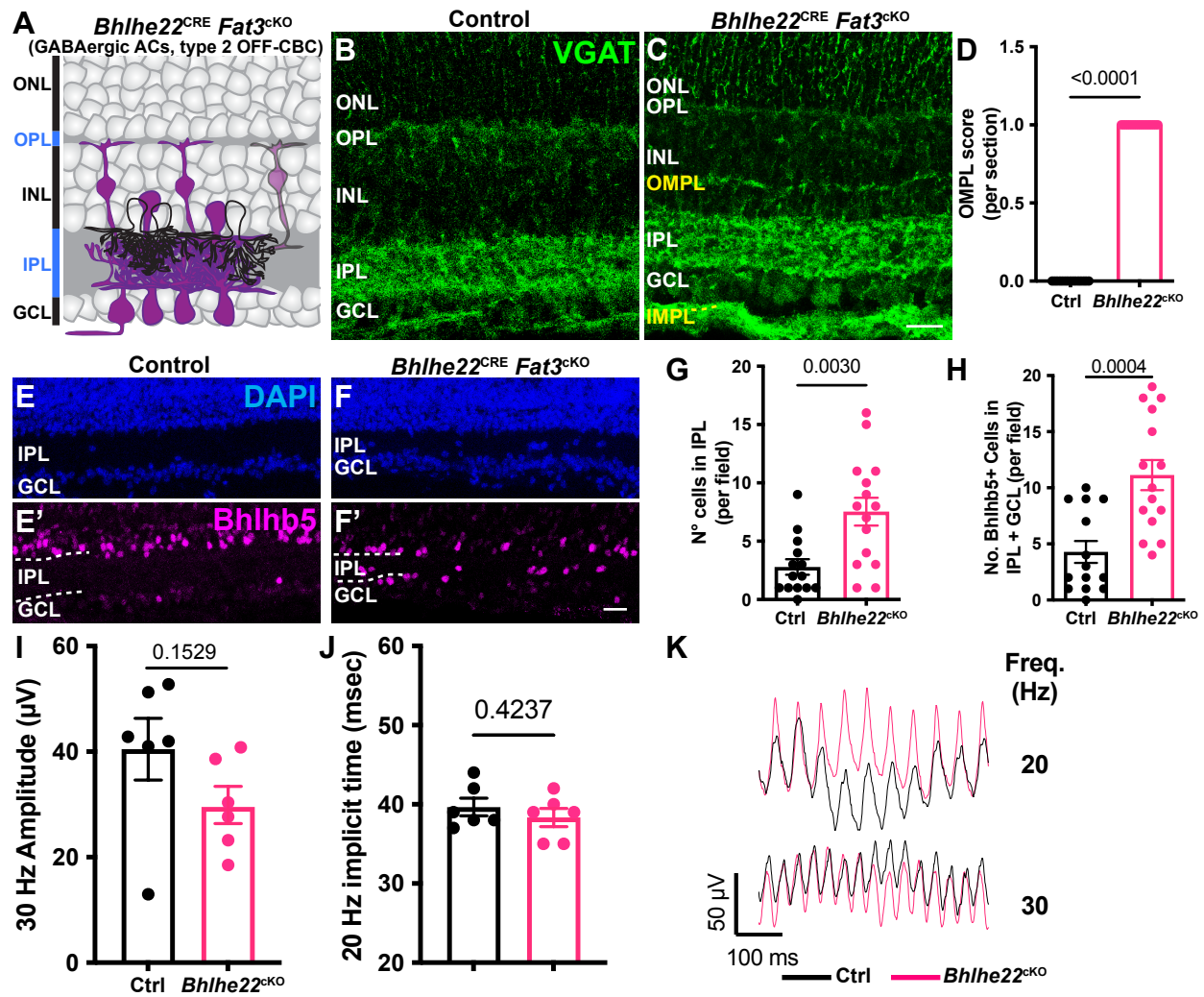

Figure S4

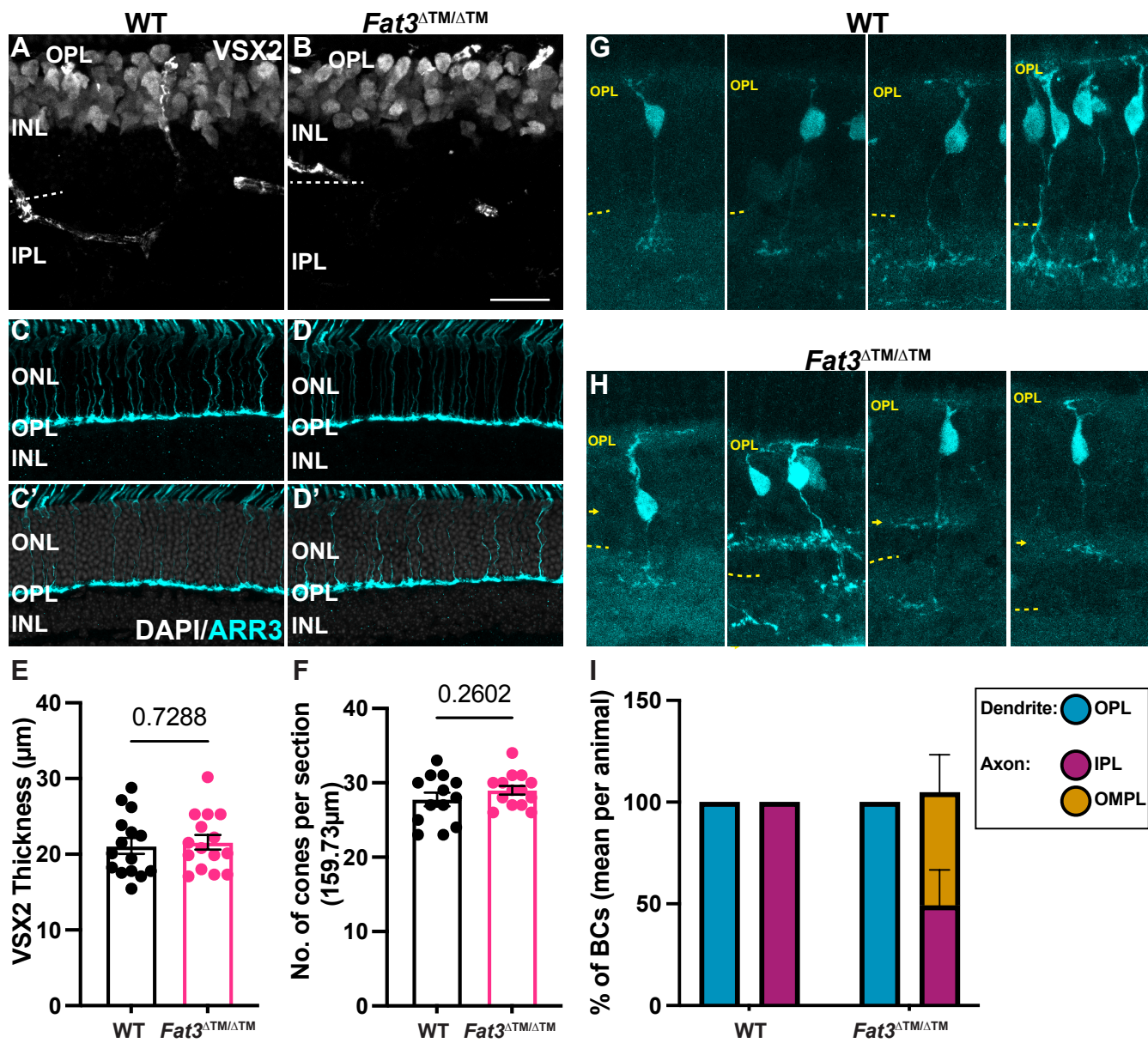

Figure S5

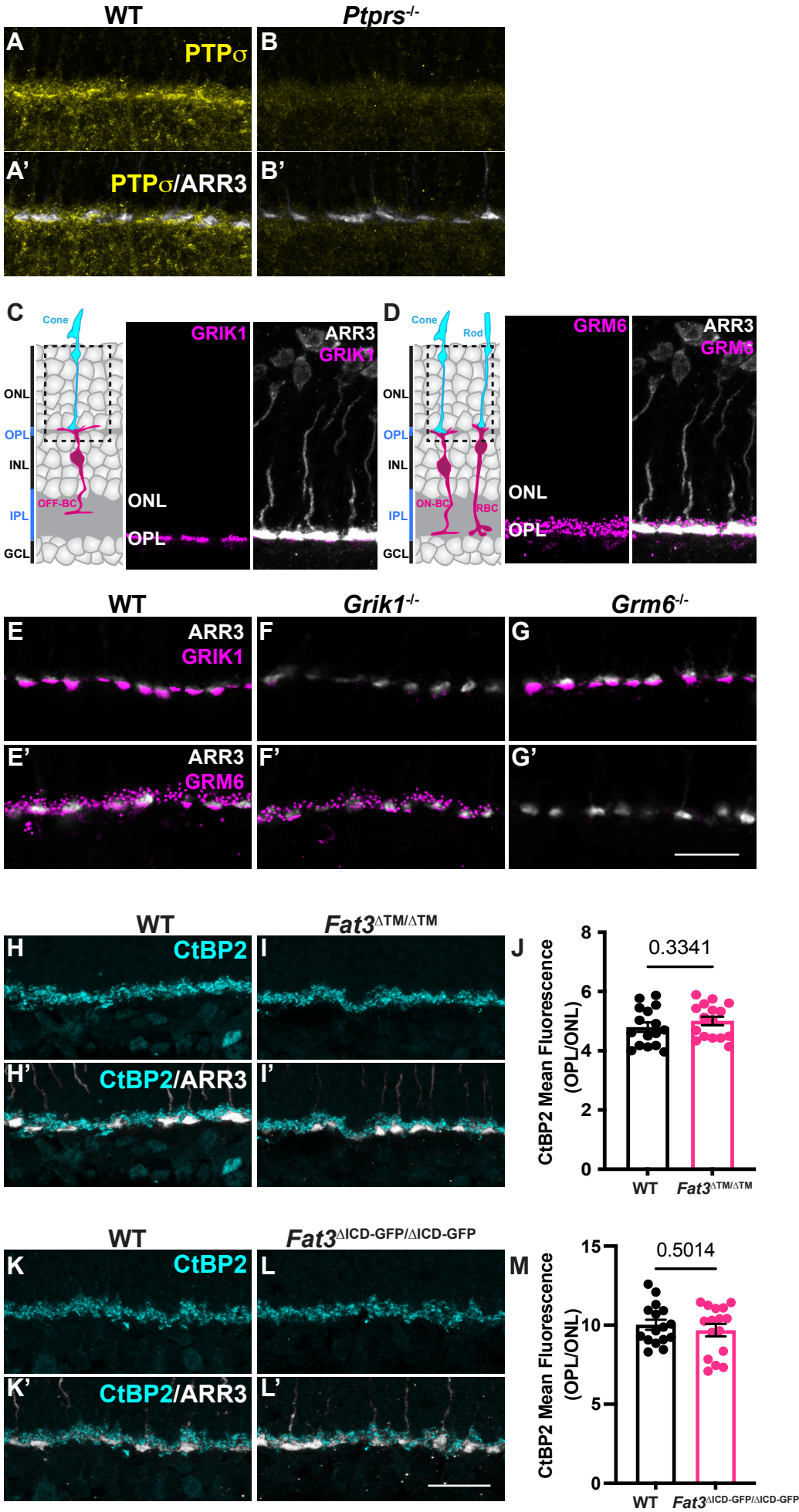

Figure S6

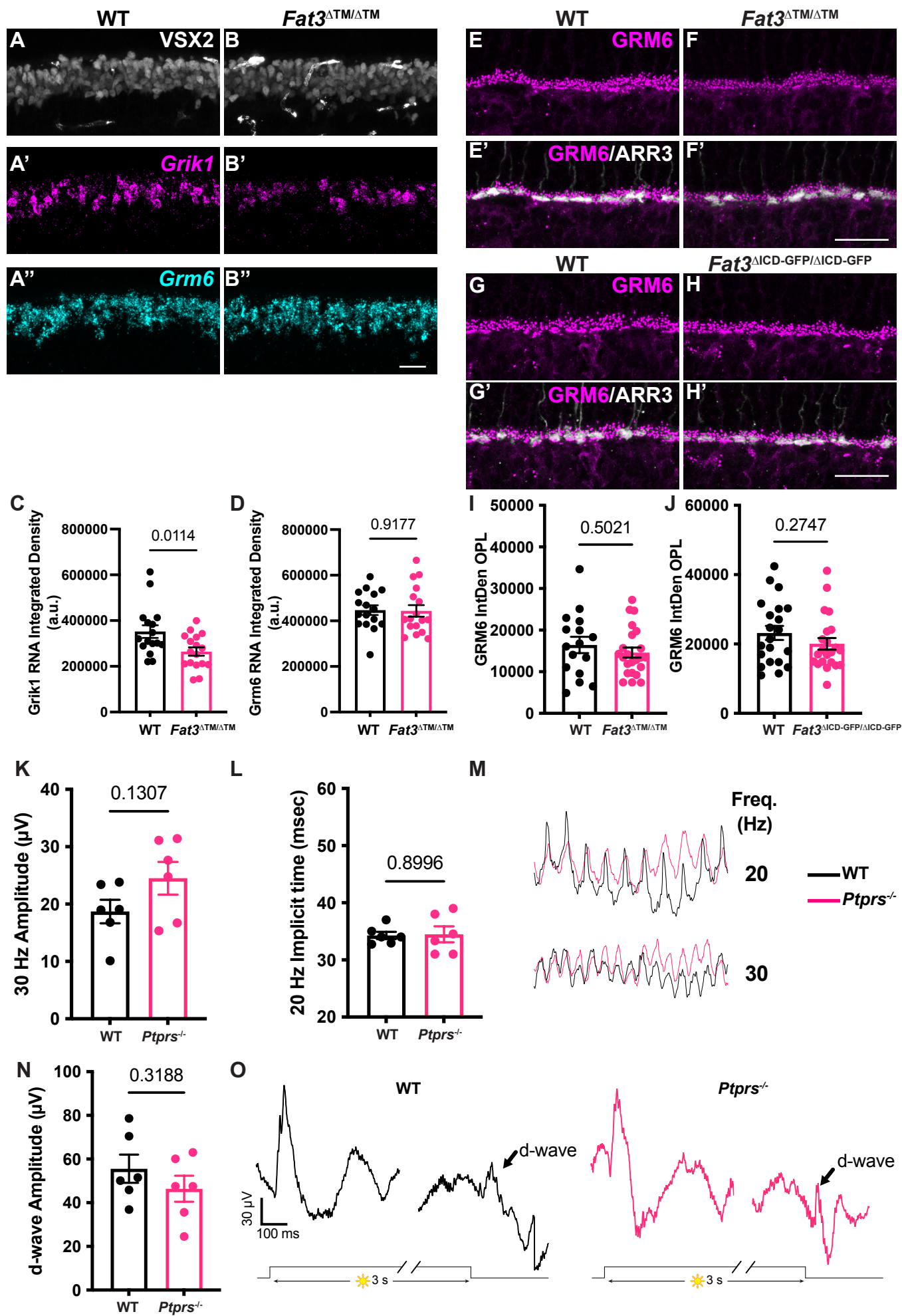

Supplement: Supplement 1 — Figure S1 (related to Figure 1): Scotopic and photopic ERG of FAT3-deficient mice. A. Representative OCT images of Fat3Δ™/+ control and Fat3Δ™/Δ™ eyes. B. Representative scotopic ERG raw traces of Fat3Δ™/+ and Fat3Δ™/Δ™ eyes elicited by 0.1 cd s/m2 flashes. C. Statistics of scotopic ERG parameters (amplitude and implicit time of a-wave and b-wave) of Fat3Δ™/+ (n=8) and Fat3Δ™/Δ™ (n=10) eyes. D. Representative photopic ERG raw traces of Fat3+/− and Fat3−/− eyes elicited by 1, 10, 100 and 1,000 cd s/m2 flashes at 30 cd/m2 background light to saturate the response from rod-pathway. E. Ensemble-averaged photopic ERG b-wave amplitude from Fat3Δ™/+ (n=8) and Fat3Δ™/Δ™ (n=10) eyes. F. Schematics of fear conditioning and optomotor behavioral experiment. On Day 1, a mouse is brought to the electric-shock cage with a floor of metal bars for habituation of the environment. On Day 2, the mouse is conditioned by electrical shock paired with 33 Hz flashing light. On Day 3 (see Supplementary movies for representative recordings from Fat3 mutant mice), the mouse is first subjected to a contextual check, in which the “Context” measures the freezing time of the mouse after it is brought back to the electric shock cage, which presents a fear-associated context environment, without the shock. “Static” measures the freezing time of the mouse with a static light, after the covering the metal bars and an odor change. Following this measurement, a 33 Hz flickering light is turned on, and the freezing time of the mouse is measured, as the “flicker” time. G. Fear conditioning responses as freezing time (sec) from Fat3Δ™/+ (n=8) and Fat3Δ™/Δ™ (n=9) mice. One-way ANOVA with Dunnett multiple comparison test. H. The visual threshold of spatial frequency of WT (n=8) and Fat3Δ™/Δ™ (n=10) mice measured with the optomotor behavioral assay. Unpaired two-tailed Student’s t test. Abbreviations: RPE, retinal pigmented epithelium; IS/OS, inner-outer segments junction; OLM, outer limiting membrane; OPL, o [file media-1.pdf]
