# Supplementary material for: High temporal frequency light response in mouse retina is mediated by ON and OFF bipolar cells and requires FAT3 signaling
Source: bioRxiv. 2023 Nov 4:2023.11.02.565326. Preprint. [Version 1] doi: 10.1101/2023.11.02.565326 (PMC10635074; doi:10.1101/2023.11.02.565326)
Supplement: Supplementary file 1 [file media-1.pdf]

**Figure S1**

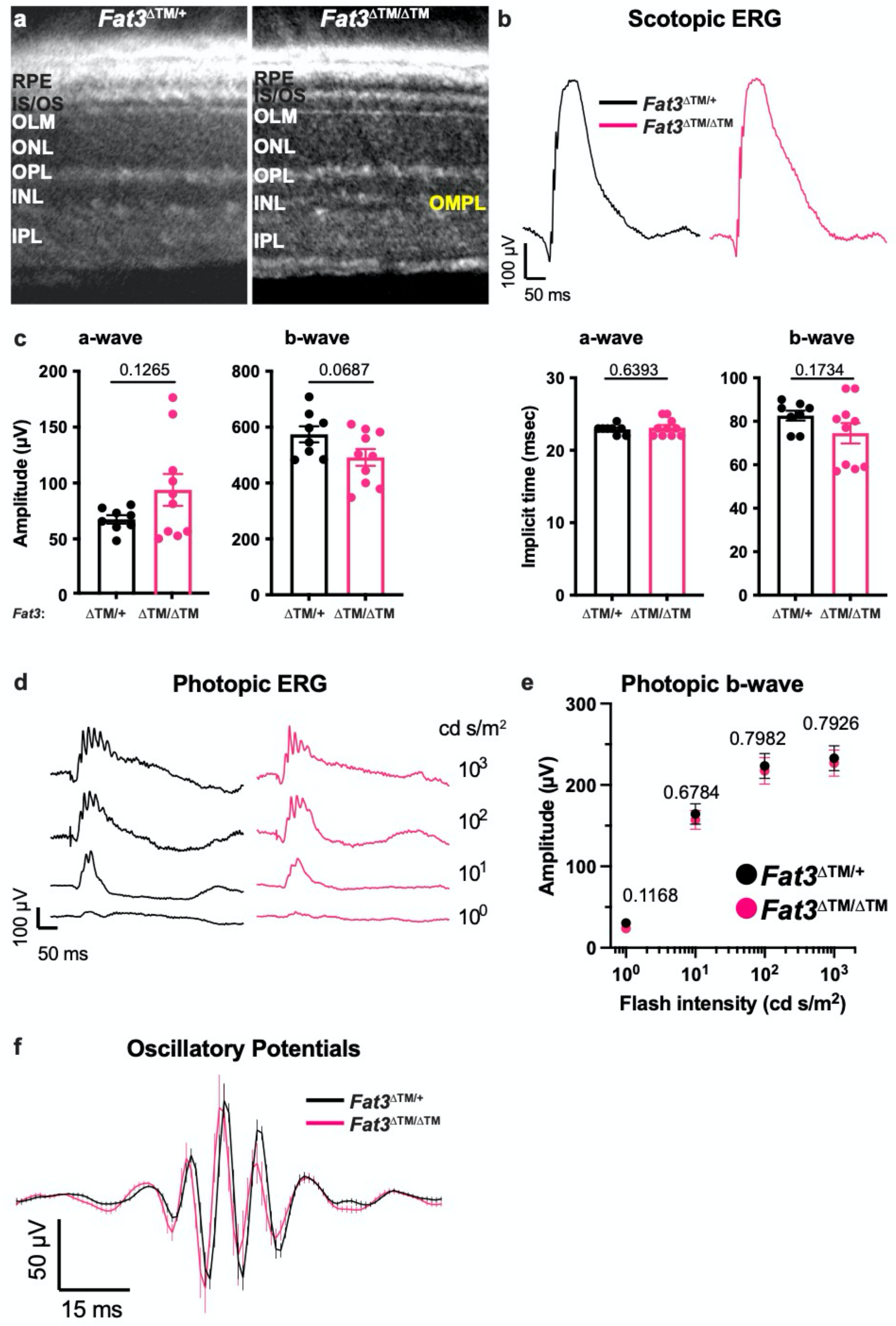


<u>**Figure S1 (related to Figure 1 and 2):**</u> **Scotopic and photopic ERG of FAT3-deficient mice.**

(a) Representative OCT images of *Fat3*$^{\Delta TM/+}$ control and *Fat3*$^{\Delta TM/\Delta TM}$ eyes.

(b) Representative scotopic ERG raw traces of *Fat3*$^{\Delta TM/+}$ and *Fat3*$^{\Delta TM/\Delta TM}$ eyes elicited by 0.1 cd s/m$^2$ flashes.

(c) Statistics of scotopic ERG parameters (amplitude and implicit time of a-wave and b-wave) of *Fat3*$^{\Delta TM/+}$ (n=8) and *Fat3*$^{\Delta TM/\Delta TM}$ (n=10) eyes.

(d) Representative photopic ERG raw traces of *Fat3*$^{+/-}$ and *Fat3*$^{-/-}$ eyes elicited by 1, 10, 100 and 1,000 cd s/m$^2$ flashes at 30 cd/m$^2$ background light to saturate the response from rod-pathway.

(e) Ensemble-averaged photopic ERG b-wave amplitude from *Fat3*$^{\Delta TM/+}$ (n=8) and *Fat3*$^{\Delta TM/\Delta TM}$ (n=10) eyes.

(f) Averaged Oscillatory potentials of *Fat3*$^{\Delta TM/+}$ (n=8) and *Fat3*$^{\Delta TM/\Delta TM}$ (n=10) eyes at scotopic condition elicited by 0.1 cd s/m$^2$ flashes (same as Figure S1B).

Abbreviations: RPE, retinal pigmented epithelium; IS/OS, inner-outer segments junction; OLM, outer limiting membrane; OPL, outer plexiform layer; IPL, inner plexiform layer; IMPL: inner misplaced plexiform layer. NS: non-significant. All data are presented as mean ± SEM.

**Figure S2**

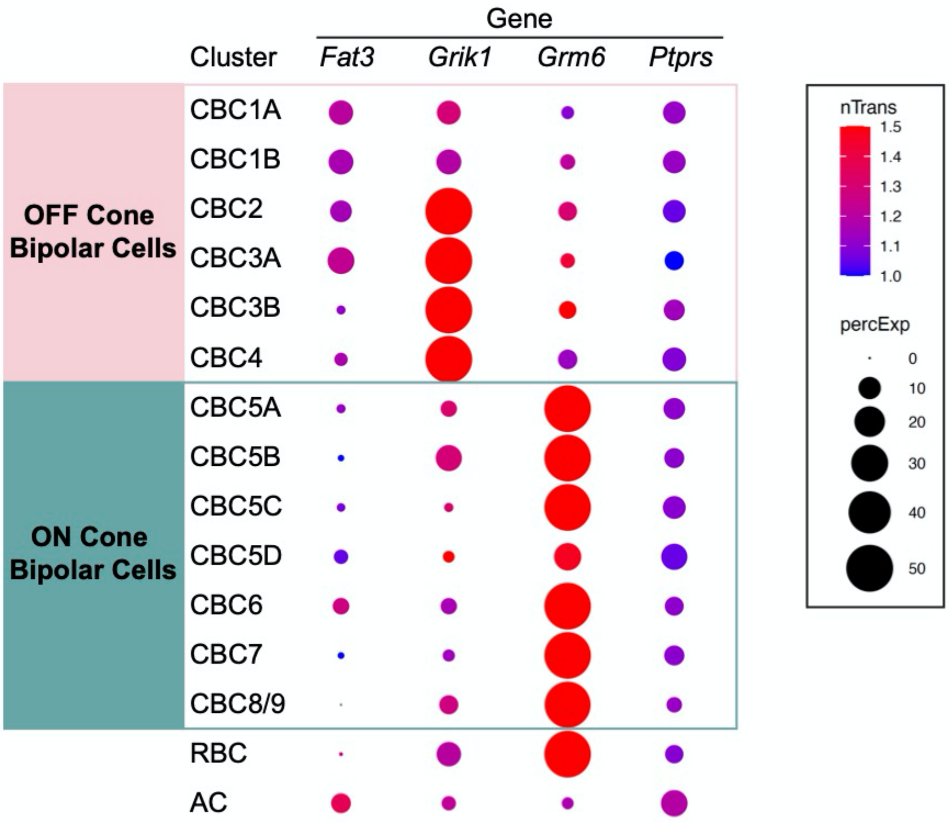


**<u>Figure S2 (related to Figure 2)</u>: scRNAseq data display.** Expression of *Fat3*, *Grik1*, *Grm6* and *Ptprs* gene transcripts by bipolar cell identity are displayed. These data is based on Shekhar et al dataset[1].

Figure S3

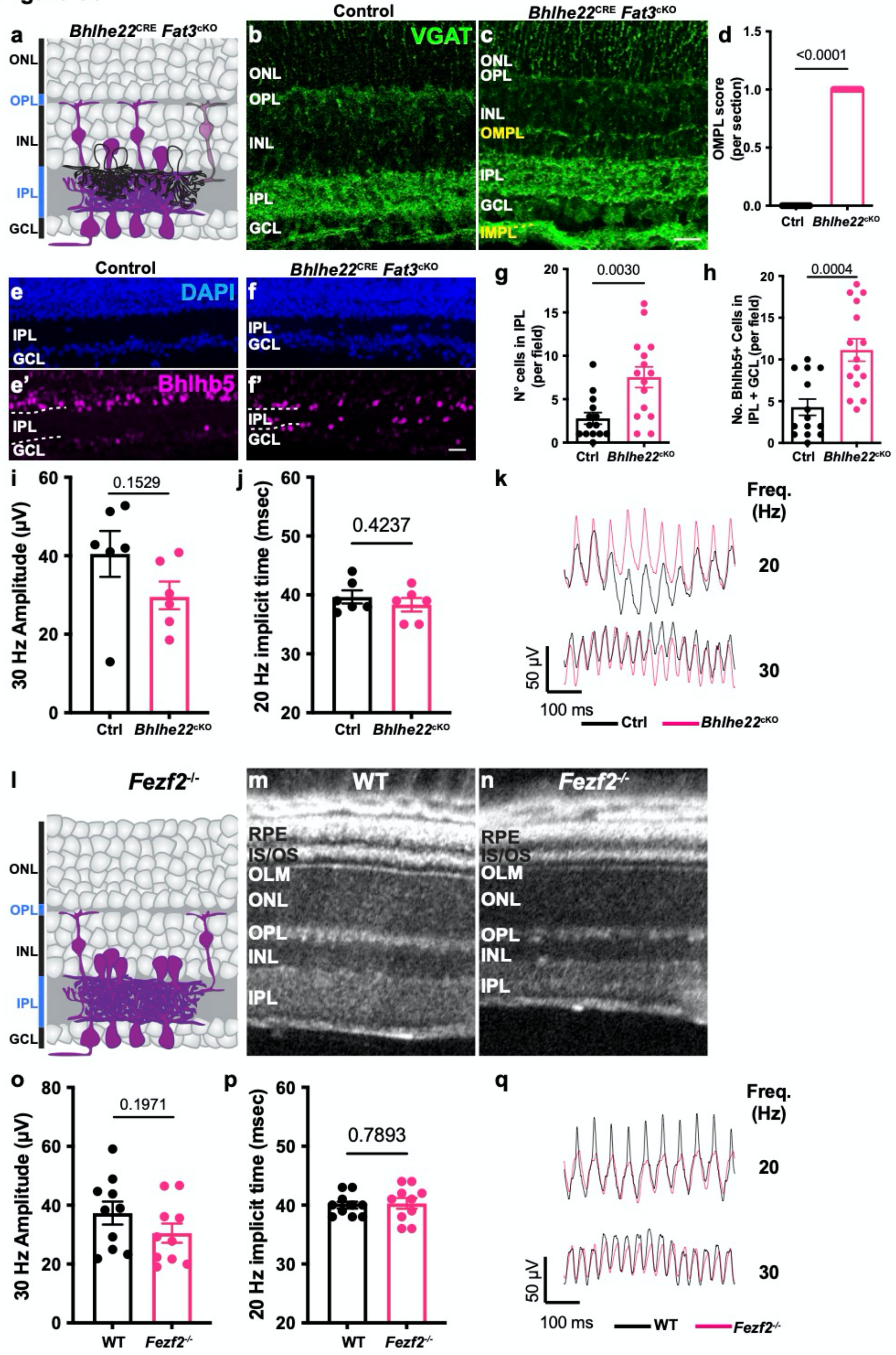


**<u>Figure S3 (related to Figure 3)</u>: Effect of removal of *Fat3* from OFF-CBC type 2 on flicker ERG.**

(a) Schematic representation of cells types, i.e. type 2 OFF-CBCs and GABAergic ACs, that lose *Fat3* expression in a *Bhlhe22*$^{CRE}$ cKO. Magenta coloring represents cell type expression of Fat3.

(b) VGAT immunostaining for control *Bhlhe22*$^{CRE/+}$;*Fat3*$^{fl/+}$ mice.

(c) VGAT immunostaining for *Bhlhe22*$^{CRE/+}$;*Fat3*$^{fl/\Delta TM}$ cKOs.

(d) Quantification of the OMPL score for *Bhlhe22*$^{CRE}$ *Fat3*cKO. Controls (*Bhlhe22*$^{CRE/+}$;*Fat3*$^{fl/+}$): 0.0 ± 0.0 (n=15 sections, N=3 animals); *Bhlhe22*$^{cKO}$ (*Bhlhe22*$^{CRE/+}$;*Fat3*$^{fl/\Delta TM}$): 1.0 ± 0.0 (n=18 sections, N=3 animals), Mann-Whitney test.

(e) DAPI and Bhlhb5 (a.k.a. *Bhlhe22* gene encoded protein) staining of *Bhlhe22*$^{CRE/+}$;*Fat3*$^{fl/+}$ mouse retinas.

(f) DAPI and Bhlhb5 staining of *Bhlhe22*$^{CRE/+}$;*Fat3*$^{fl/\Delta TM}$ retinas.

(g) Quantification of the number of nuclei per field in the IPL. Controls (*Bhlhe22*$^{CRE/+}$;*Fat3*$^{fl/+}$): 2.79 ± 0.66 (n=14 sections, N=3 animals); *Bhlhe22*$^{cKO}$ (*Bhlhe22*$^{CRE/+}$;*Fat3*$^{fl/\Delta TM}$): 7.53 ± 1.20 (n=15 sections, N=3 animals). Mann-Whitney test.

(h) Quantification of the number of Bhlhb5+ nuclei per field in the IPL and GCL. Controls (*Bhlhe22*$^{CRE/+}$;*Fat3*$^{fl/+}$): 4.29 ± 0.98 (n=14 sections, N=3 animals); *Bhlhe22*$^{cKO}$ (*Bhlhe22*$^{CRE/+}$;*Fat3*$^{fl/\Delta TM}$): 11.13 ± 1.34 (n=15 sections, N=3 animals). *t* test.

(i) Flicker ERG amplitude at 30 Hz for controls (*Bhlhe22*$^{CRE/+}$;*Fat3*$^{fl/+}$, n=6 eyes) and *Bhlhe22*$^{CRE}$ *Fat3*$^{cKO}$ (*Bhlhe22*$^{CRE/+}$;*Fat3*$^{fl/\Delta TM}$, n=6 eyes).

(j) Flicker ERG implicit time at 20 Hz for controls (*Bhlhe22*$^{CRE/+}$;*Fat3*$^{fl/+}$, n=6 eyes) and *Bhlhe22*$^{CRE}$ *Fat3*$^{cKO}$ (*Bhlhe22*$^{CRE/+}$;*Fat3*$^{fl/\Delta TM}$, n=6 eyes).

(k) Representative flicker ERG raw traces for controls (*Bhlhe22*$^{CRE/+}$;*Fat3*$^{fl/+}$, n=6 eyes) and *Bhlhe22*$^{CRE}$ *Fat3*$^{cKO}$ (*Bhlhe22*$^{CRE/+}$;*Fat3*$^{fl/\Delta TM}$, n=6 eyes) at 20 and 30 Hz.

(l) Schematic representation of cells types, i.e. all OFF-CBCs except for type 2, that are lost in a *Fezf2*$^{-/-}$ mutant retinas. Magenta coloring represents cell type expression of Fat3.

(m) Representative OCT images of WT control eyes.

(n) Representative OCT images of *Fezf2*$^{-/-}$ eyes.

(o) Flicker ERG amplitude at 30 Hz for WT (n=10 eyes) and *Fezf2*$^{-/-}$ retinas (n=10 eyes).

(p) Flicker ERG implicit time at 20 Hz for WT (n=10 eyes) and *Fezf2*$^{-/-}$ retinas (n=10 eyes).

(q) Representative flicker ERG raw traces for WT (n=10 eyes) and *Fezf2*$^{-/-}$ retinas (n=10 eyes) at 20 and 30 Hz.

Scale bars: 20μm.

Figure S4

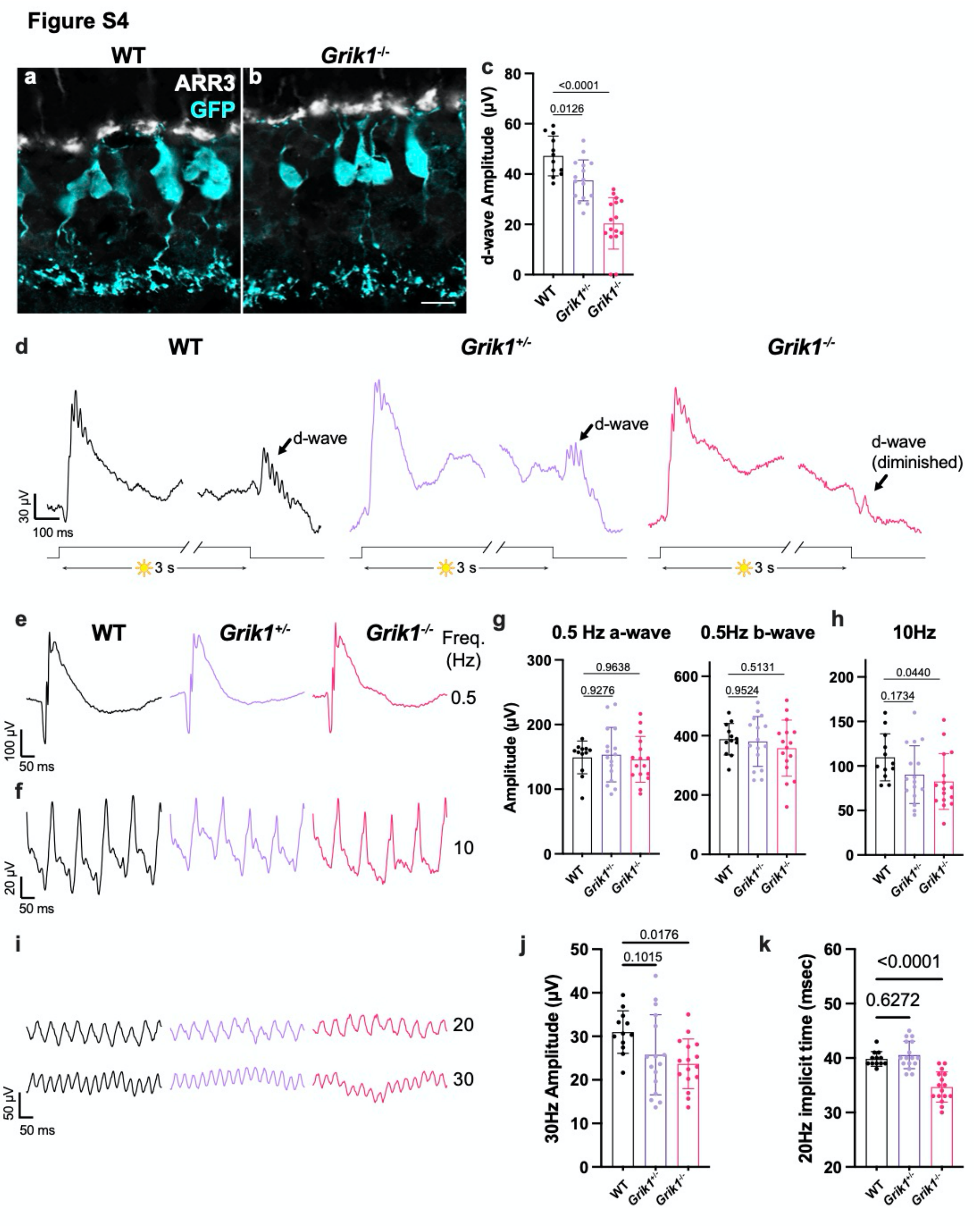


**<u>Figure S4 (related to Figure 5)</u>: Flicker ERG, step ERG and OFF cone bipolar cell morphology of GRIK1-deficient mice.**

(a) Immunostaining of retina cross sections from adult WT mice that received AAV8-Grik1-GFP at P2 (white: ARR3, cyan: GFP).

(b) Immunostaining of retina cross sections from adult *Grik1*$^{-/-}$ mice.

(c) Statistics of step ERG d-wave amplitudes of WT, *Grik1*$^{+/-}$ and *Grik1*$^{-/-}$ eyes elicited by a 3-second step of light at 1000 cd/m$^2$ intensity.

(d) Representative step ERG raw traces of WT, *Grik1*$^{+/-}$ and *Grik1*$^{-/-}$ eyes elicited by a 3-second step light at 1000 cd/m$^2$ intensity.

(e) Representative flicker ERG raw traces of WT, *Grik1*$^{+/-}$ and *Grik1*$^{-/-}$ eyes at 0.5 Hz frequencies.

(f) Representative flicker ERG raw traces of WT, *Grik1*$^{+/-}$ and *Grik1*$^{-/-}$ eyes at 10 Hz frequencies.

(g) Ensemble-averaged flicker 0.5 Hz ERG amplitudes (a- and b-waves) from WT, *Grik1*$^{+/-}$ and *Grik1*$^{-/-}$ eyes.

(h) Ensemble-averaged flicker 10 Hz ERG amplitude (bottom-to-peak) from WT, *Grik1*$^{+/-}$ and *Grik1*$^{-/-}$ eyes.

(i) Representative flicker ERG raw traces of WT, *Grik1*$^{+/-}$ and *Grik1*$^{-/-}$ eyes elicited by 3.162 cd s/m$^2$ flashes at 20 and 30 Hz frequencies.

(j) Flicker ERG amplitude at 30 Hz for WT, *Grik1*$^{+/-}$ and *Grik1*$^{-/-}$ eyes. P values: *Grik1*$^{-/-}$ vs. WT.

(k) Flicker ERG implicit time at 20 Hz for WT, *Grik1*$^{+/-}$ and *Grik1*$^{-/-}$ eyes.

Figure S5

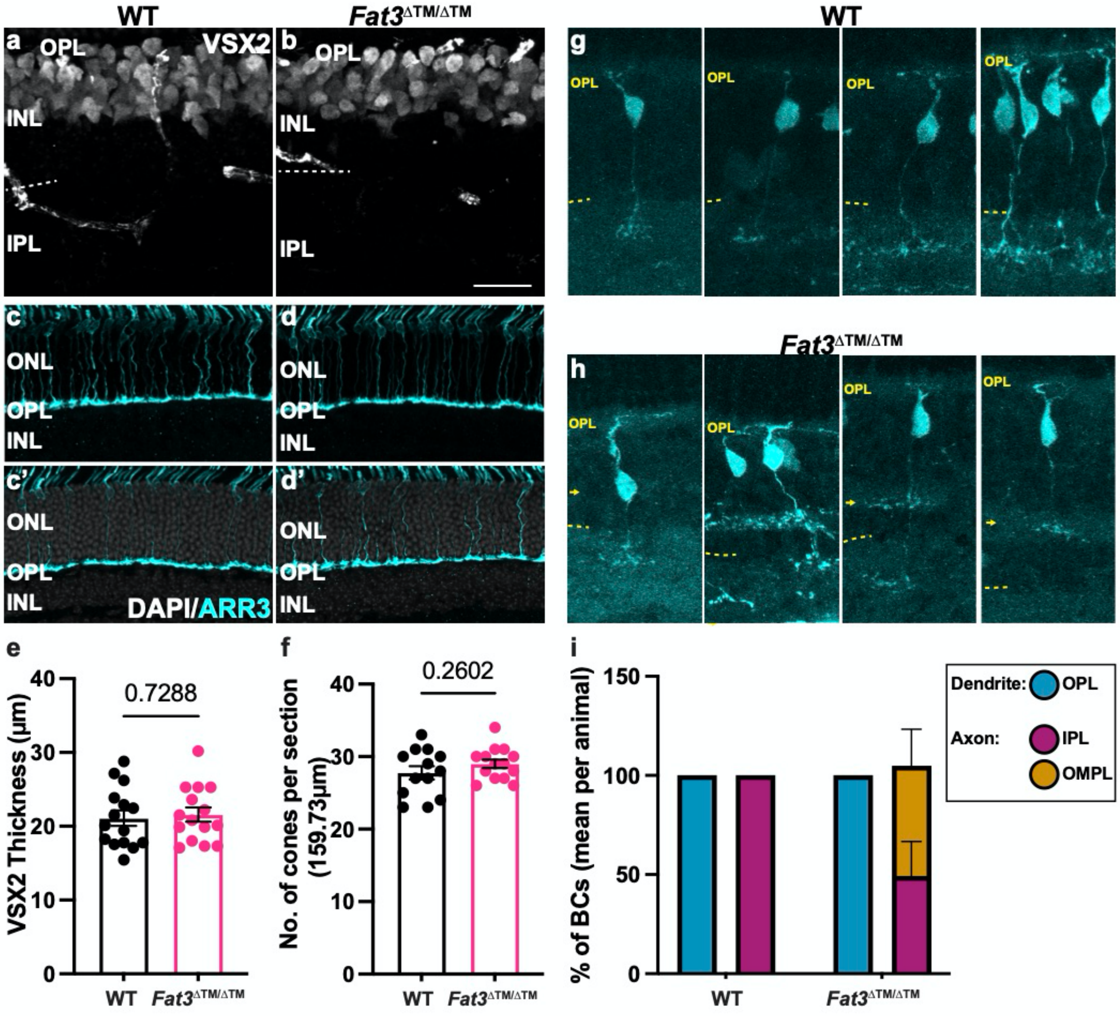


**Figure S5 (related to Figure 6): BC and cones numbers, visualization of *Grik1*+ BCs morphology via injection of an AVV-Grik1-GFP virus in *Fat3* mutants.**

(a) VSX2 immunostaining of WT retinas.

(b) VSX2 immunostaining of *Fat3*$^{\Delta TM/\Delta TM}$ retinas.

(c) ARR3 immunohistochemistry of WT retinas. c' shows ARR3 staining together with DAPI staining.

(d) ARR3 immunohistochemistry of *Fat3*$^{\Delta TM/\Delta TM}$ retinas. d' shows ARR3 staining together with DAPI staining.

(e) Quantification of thickness of area occupied by VSX2 staining, as shown in d and e. WT controls: 21.09 ± 1.045 (n=15 sections, N=4 animals); *Fat3*$^{\Delta TM/\Delta TM}$: 21.59 ± 0.969 (n=15 sections, N=4 animals). t test.

(f) Quantification of number of cones marked by ARR3, as shown in f and g. WT controls: 27.77 ± 0.907 (n=13 sections, N=4 animals); *Fat3*$^{\Delta TM/\Delta TM}$: 29.00 ± 0.593 (n=14 sections, N=4 animals). t test.

(g) Examples of retinal sections showing cells expressing GFP under the control of the *Grik1* enhancer. The GFP reporter was introduced through *in vivo* injection of AAV8-Grik1-GFP. Wild type (WT) retinas are shown in g.

(h) *Fat3*$^{\Delta TM/\Delta TM}$ retinas injected with AAV8-Grik1-GFP. The OPL is indicated with a yellow arrowhead and the OMPL, labeled with VGAT staining, is marked with a yellow arrow. Scale bar: 20μm.

(i) Quantification of *Grik1*+ BC dendrites in the OPL (WT Controls: 100%; *Fat3*$^{\Delta TM/\Delta TM}$: 100%), axons in the IPL (WT Controls: 100%; *Fat3*$^{\Delta TM/\Delta TM}$: 49 ± 17.4%), and axons in the OMPL (WT Controls: 0%; *Fat3*$^{\Delta TM/\Delta TM}$: 56 ± 18.5%). Controls: n=5 animals, 12 cells, *Fat3*$^{\Delta TM/\Delta TM}$: n=7 animals, 22 cells. Mann-Whitney test.

Figure S6

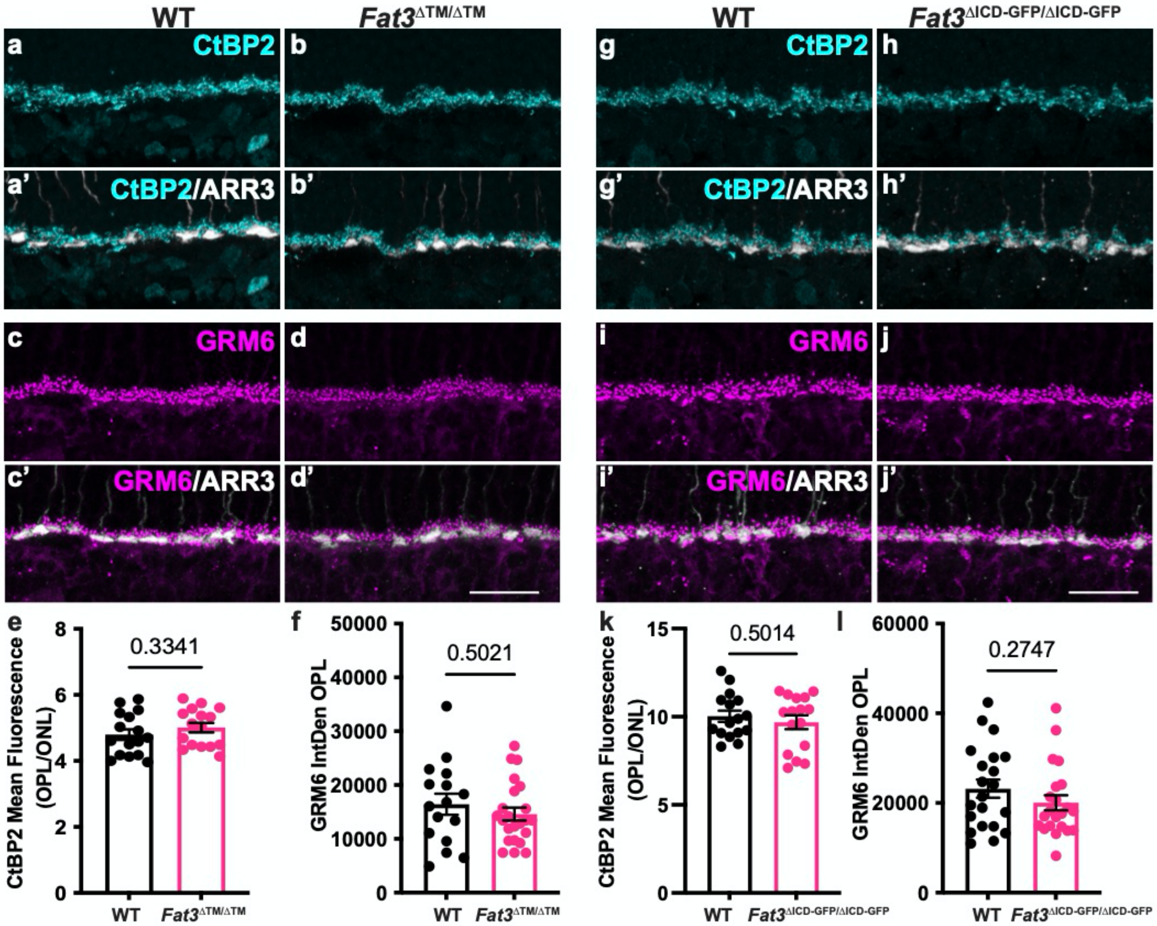


**<u>Figure S6 (related to Figure 7)</u>: Immunostaining of GRM6 and CtBP2 upon loss of *Fat3* in mouse retina.**

(a) Immunostaining for CtBP2, a marker of presynaptic ribbon in WT retinas.

(b) Immunostaining for CtBP2 *Fat3*$^{\Delta TM/\Delta TM}$ retinas. Cone arrestin (ARR3) labels the cone photoreceptors endings in the OPL in a' and b'.

(c) Immunostaining for GRM6 in WT retinas.

(d) Immunostaining for GRM6 in *Fat3*$^{\Delta TM/\Delta TM}$ retinas. Cone arrestin (ARR3) labels the cone photoreceptors endings in the OPL in c' and d'.

(e) Quantification of CtBP2 mean fluorescence intensity in the OPL, normalized by ONL signal. Wild type (WT) Controls: 4.8 ± 0.16 (n=16 sections, N=4 animals); *Fat3*$^{\Delta TM/\Delta TM}$: 5.0 ± 0.14 (n=16 sections, N=4 animals), *t* test.

(f) Quantification of GRM6 integrated intensity in the OPL. WT Controls: 16435 ± 1953 (n=16 sections, N=4 animals); *Fat3*$^{\Delta TM/\Delta TM}$: 14603 ± 1195 (n=23 sections, N=5 animals), Mann-Whitney test.

(g) Immunostaining for CtBP2 in WT retinas, littermates of *Fat3*$^{\Delta ICD\text{-}GFP/\Delta ICD\text{-}GFP}$ animals.

(h) Immunostaining for CtBP2 in *Fat3*$^{\Delta ICD\text{-}GFP/\Delta ICD\text{-}GFP}$ retinas. Cone arrestin (ARR3) labels the cone photoreceptors endings in the OPL in g' and h'.

(i) Immunostaining for GRM6 in WT retinas, littermates of *Fat3*$^{\Delta ICD\text{-}GFP/\Delta ICD\text{-}GFP}$ animals.

(j) Immunostaining for GRM6 in *Fat3*$^{\Delta ICD\text{-}GFP/\Delta ICD\text{-}GFP}$ retinas. Cone arrestin (ARR3) labels the cone photoreceptors endings in the OPL in i' and j'.

(k) Quantification of CtBP2 mean fluorescence intensity in the OPL, normalized by ONL signal. Wild type (WT) Controls: 10.03 ± 0.32 (n=16 sections, N=4 animals); *Fat3*$^{\Delta ICD/\Delta ICD}$: 9.7 ± 0.39 (n=16 sections, N=4 animals), *t* test.

(l) Quantification of GRM6 integrated intensity in the OPL. WT Controls: 23190 ± 2018 (n=21 sections, N=5 animals); *Fat3*$^{\Delta ICD/\Delta ICD}$: 20051 ± 1696 (n=22 sections, N=5 animals), Mann-Whitney test.

**Figure S7**

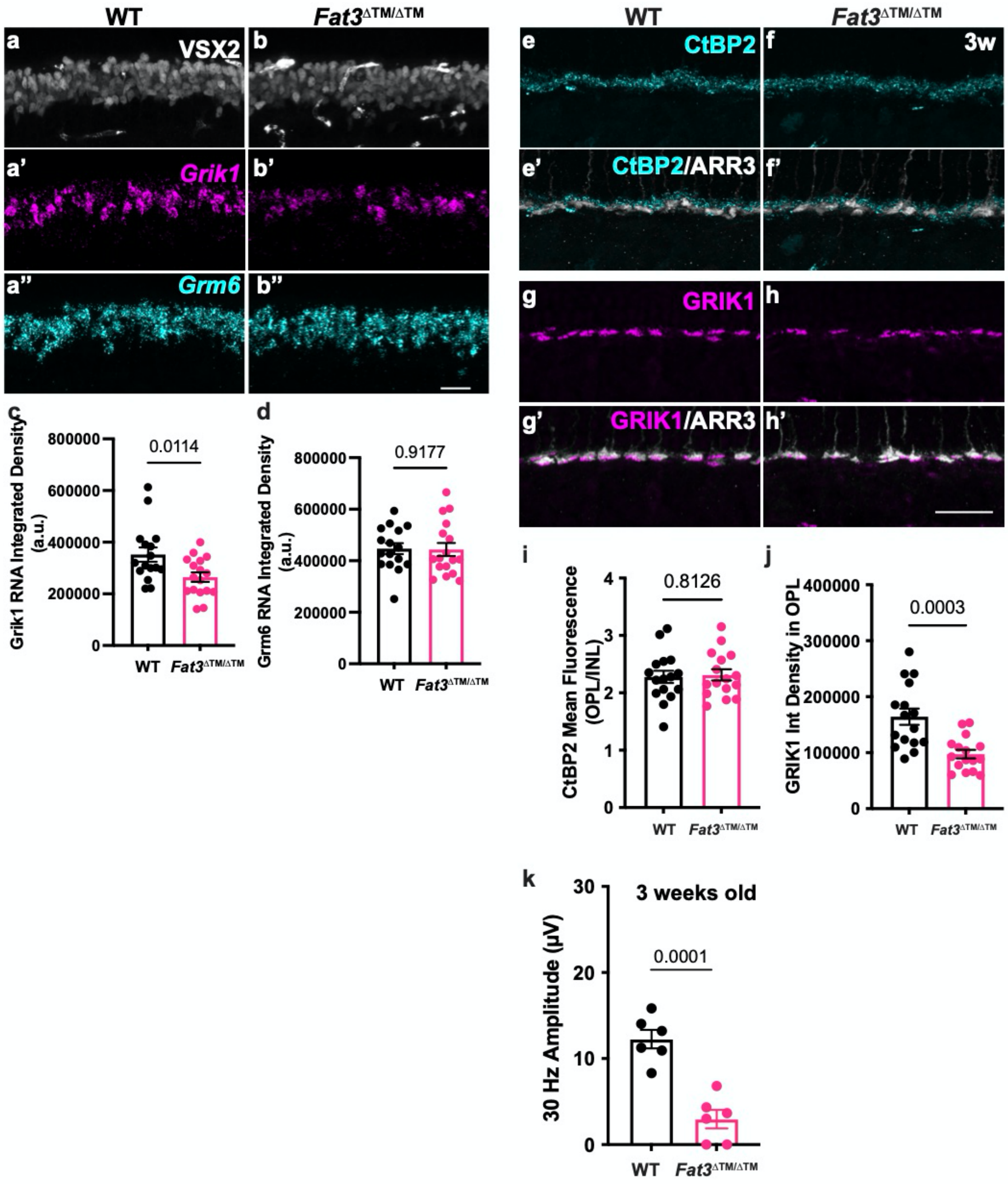


**<u>Figure S7 (related to Figure 7)</u>: Immunostaining of GRIK1 and CtBP2 in the retina upon loss of *Fat3* at earlier ages.**

(a) VSX2 immunostaining (a) after *in situ* hybridization for *Grik1* (a') and *Grm6* (a'') in WT retina.

(b) VSX2 immunostaining (b) after *in situ* hybridization for *Grik1* (b') and *Grm6* (b'') in *Fat3*$^{\Delta TM / \Delta TM}$ retina.

(c) Quantification of *Grik1* RNA pixel density in bipolar cells. WT: 352076 ± 27459, n=16 sections, N= 4 animals; *Fat3*$^{\Delta TM / \Delta TM}$: 264572 ± 18120, n=17 sections, N=4 animals. t test.

(d) Quantification of *Grm6* RNA pixel density in bipolar cells. WT: 447054 ± 21371, n=16 sections, N= 4 animals; *Fat3*$^{\Delta TM / \Delta TM}$: 443575 ± 25398, n=17 sections, N=4 animals. t test.

(e) Immunostaining for CtBP2, a marker of presynaptic ribbon in WT.

(f) Immunostaining for CtBP2, a marker of presynaptic ribbon in *Fat3*$^{\Delta TM / \Delta TM}$ retinas at 3 weeks. Cone arrestin (ARR3) labels the cone photoreceptors endings in the OPL in e' and f'.

(g) Immunostaining for GRIK1 in WT retinas.

(h) Immunostaining for GRIK1 in *Fat3*$^{\Delta TM / \Delta TM}$ retinas. Cone arrestin (ARR3) labels the cone photoreceptors endings in the OPL in c' and d' at 3 weeks of age.

(i) Quantification of CtBP2 mean fluorescence intensity in the OPL, normalized by ONL signal. Wild type (WT) Controls: 2.28 ± 0.11 (n=16 sections, N=4 animals); *Fat3*$^{\Delta TM / \Delta TM}$: 2.31 ± 0.10 (n=16 sections, N=4 animals), *t* test.

(j) Quantification of GRIK1 integrated intensity in the OPL. WT Controls: 164356 ± 14416 (n=16 sections, N=4 animals); *Fat3*$^{\Delta TM / \Delta TM}$: 97502 ± 7532 (n=16 sections, N=4 animals), *t* test.

(k) Flicker ERG amplitude at 30 Hz for *Fat3*$^{\Delta TM / \Delta TM}$ retinas (n=6 eyes for control and mutants) at 3 weeks of age.

Scale bars: 20μm.

Figure S8

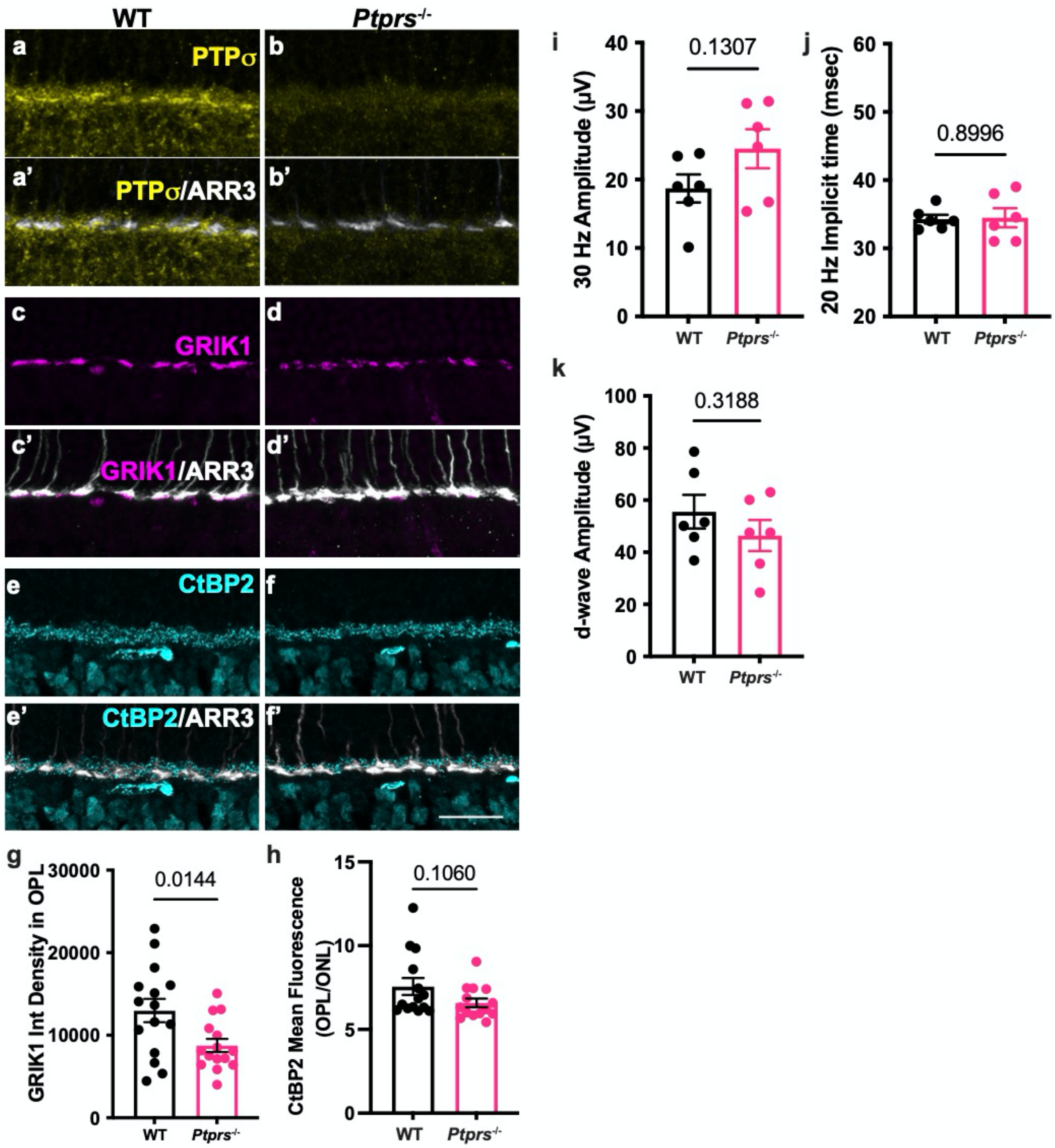


**<u>Figure S8 (related to Figure 7)</u>: Immunostaining of GRM6 and CtBP2 upon loss of *Fat3* in mouse retina.**

(a) PTPσ immunostaining in WT retinas

(b) PTPσ immunostaining in *Ptprs*$^{-/-}$ retinas. Cone arrestin (ARR3) labels the cone photoreceptors endings in the OPL in a' and b'.

(c) Immunostaining for GRIK1 in WT retinas.

(d) Immunostaining for GRIK1 in *Ptprs*$^{-/-}$ retinas. Cone arrestin (ARR3) labels the cone photoreceptor endings in the OPL in c' and d'.

(e) Immunostaining for CtBP2 in WT retinas.

(f) Immunostaining for CtBP2 in *Ptprs*$^{-/-}$ retinas. Cone arrestin (ARR3) labels the cone photoreceptors endings in the OPL in e' and f'.

(g) Quantification of GRIK1 integrated intensity in the OPL. WT Controls: 12996 ± 1418 (n=15 sections, N=3 animals); *Ptprs*$^{-/-}$: 8758 ± 792 (n=15 sections, N=3 animals), t test.

(h) Quantification of CtBP2 mean fluorescence intensity in the OPL, normalized by ONL signal. Wild type (WT) Controls: 7.56 ± 0.51 (n=15 sections, N=3 animals); *Ptprs*$^{-/-}$: 6.68 ± 0.26 (n=15 sections, N=3 animals), Mann-Whitney test.
Scale bar: 20μm.

(i) Flicker ERG amplitude at 30 Hz for WT (n=6 eyes) and *Ptprs*$^{-/-}$ retinas (n=6 eyes).

(j) Flicker ERG implicit time at 20 Hz for WT (n=6 eyes) and *Ptprs*$^{-/-}$ retinas (n=6 eyes).

(k) Statistics of step ERG d-wave amplitudes of WT and *Ptprs*$^{-/-}$ eyes elicited by a 3-second step of light at 1000 cd/m$^2$ intensity.
